# Inositol phosphates as an overlooked phosphorous source in marine ecosystems

**DOI:** 10.1093/ismejo/wraf161

**Published:** 2025-07-11

**Authors:** Zhao-Jie Teng, Xiao-Jie Yuan, Rui Liu, Shao-Chun Xu, Xiu-Lan Chen, Yin Chen, Yu-Zhong Zhang

**Affiliations:** State Key Laboratory of Microbial Technology, Shandong University, Shandong, Qingdao 266237, China; MOE Key Laboratory of Evolution and Marine Biodiversity, State Key Laboratory of Marine Food Processing and Safety Control, Frontiers Science Center for Deep Ocean Multispheres and Earth System & College of Marine Life Sciences, Ocean University of China, Shandong, Qingdao 266003, China; Laboratory for Marine Biology and Biotechnology, Qingdao Marine Science and Technology Center & Laoshan Laboratory, Shandong, Qingdao 266237, China; State Key Laboratory of Microbial Technology, Shandong University, Shandong, Qingdao 266237, China; State Key Laboratory of Microbial Technology, Shandong University, Shandong, Qingdao 266237, China; CAS Key Laboratory of Marine Ecology and Environmental Sciences, Institute of Oceanology, Chinese Academy of Sciences, Shandong, Qingdao 266071, China; State Key Laboratory of Microbial Technology, Shandong University, Shandong, Qingdao 266237, China; Laboratory for Marine Biology and Biotechnology, Qingdao Marine Science and Technology Center & Laoshan Laboratory, Shandong, Qingdao 266237, China; MOE Key Laboratory of Evolution and Marine Biodiversity, State Key Laboratory of Marine Food Processing and Safety Control, Frontiers Science Center for Deep Ocean Multispheres and Earth System & College of Marine Life Sciences, Ocean University of China, Shandong, Qingdao 266003, China; School of Life Sciences, University of Warwick, Coventry CV4 7AL, United Kingdom; School of Biosciences, University of Birmingham, Birmingham B15 2TT, United Kingdom; MOE Key Laboratory of Evolution and Marine Biodiversity, State Key Laboratory of Marine Food Processing and Safety Control, Frontiers Science Center for Deep Ocean Multispheres and Earth System & College of Marine Life Sciences, Ocean University of China, Shandong, Qingdao 266003, China; Laboratory for Marine Biology and Biotechnology, Qingdao Marine Science and Technology Center & Laoshan Laboratory, Shandong, Qingdao 266237, China; Marine Biotechnology Research Center, State Key Laboratory of Microbial Technology, Shandong University, Shandong, Qingdao 266237, China

**Keywords:** inositol phosphates, phytate, phosphorus limitation, phytase, proteobacteria

## Abstract

Inositol phosphates, common phosphorus storage compounds that are also crucial for eukaryotic cell signaling, constitute a significant portion of dissolved organic phosphorus in coastal waters. The hydrolysis of inositol phosphates could be an important contributor to phosphorus cycling in phosphorus-limited marine ecosystems, yet this process remains poorly understood in marine contexts. In this study, we reveal substantial concentrations of inositol phosphates in marine macrophytes, including green, brown, and red algae as well as common seagrasses, suggesting that these organisms are likely major biological sources of inositol phosphates in the oceans. A comprehensive analysis of genes involved in inositol phosphates hydrolysis in global marine metagenomes and metatranscriptomes identified key roles for γ-, α-, and δ-proteobacteria, with additional contributions from Flavobacteriia. The degradation of marine inositol phosphates was predominantly mediated by alkaline β-propeller phytases, though genes associated with acidic cysteine phytases and purple acid phytases were also widely present. Community structure and functional traits linked to inositol phosphates degradation were shaped largely by stochastic processes. Further examination of enzyme activity at the protein and community levels indicated that phytate metabolism by marine microbes is likely a widespread phenomenon in the ocean. Overall, this study highlights inositol phosphates hydrolysis as an essential yet overlooked adaptation by marine microorganisms to address phosphorus limitations in ocean ecosystems.

## Introduction

**Figure 1:**
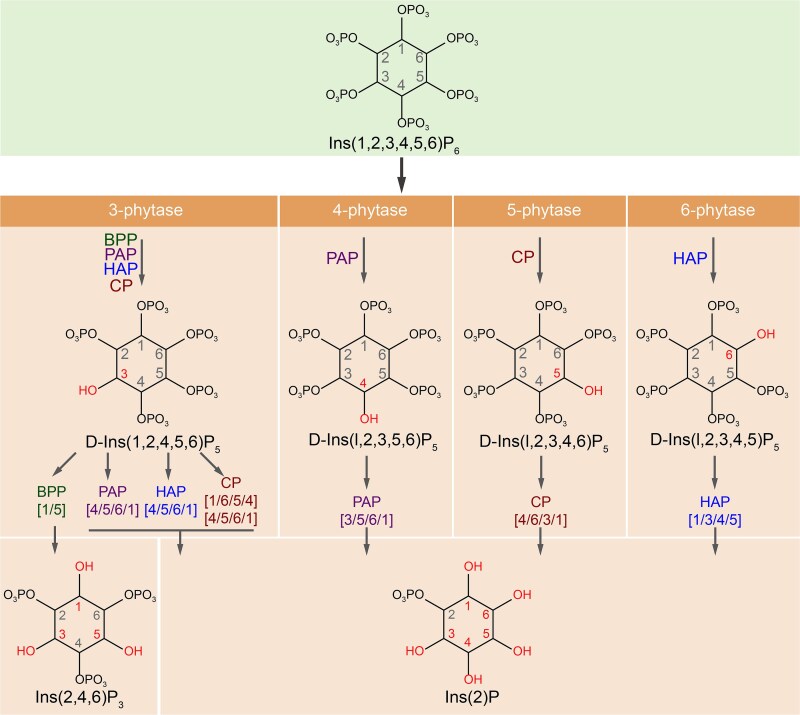
Key enzymes in phytate and other InsPs degradation. The degradation pathway of phytate and other InsPs, highlighting key intermediates involved in phosphate release. The sequential dephosphorylation sites in InsP_6_ mediated by BPP [24], HAP [19, 20], PAP [21], and CP [22, 23] are marked in red. The sequential removal of phosphate groups from InsP_5_ is indicated within square brackets. Four typical phytases BPP, PAP, HAP and CP are highlighted in green, purple, blue and brownish red, respectively, along with their corresponding dephosphorylation orders. BPP, β-propeller phytases; PAP, purple acid phytases; HAP, histidine acid phytases; CP, cysteine phytases.

Dissolved organic phosphorus (DOP) is the major reservoir of bioavailable phosphorus in the marine ecosystem that fuels primary productivity and nitrogen fixation under conditions of dissolved inorganic phosphorus (DIP) scarcity [1–4]. In marine DOP pools, phosphoesters account for over three-quarters of the total, with phosphonates and polyphosphates constituting the rest [3, 5, 6]. Compared to phosphoesters from cell structures, such as nucleic acids and phospholipids [1], inositol phosphates (InsPs, Fig. 1) serve as specialized storage forms of phosphoesters that are universally present in living organisms [7].

Phytate (*myo*-inositol-1,2,3,4,5,6-hexakisphosphate, InsP_6_) is the predominant form of InsPs [7]. Organisms have evolved efficient mechanisms to hydrolyze phytate into DIP for cell growth by utilizing phytases, a group of phosphohydrolases that initiate the stepwise removal of phosphate residues from phytate (Fig. 1) [8]. Indeed, the physiological characteristics, crystal structures, and the widely taxonomic distribution of phytases have been extensively documented previously [7–11]. Generally, phytases can be classified as β-Propeller Phytases (BPP) [12], Cysteine Phytases (CP) [13], Histidine Acid Phytases (HAP) [14], and Purple Acid Phytases (PAP) [15] based on their catalytic mechanisms (Fig. 1, Table 1), though atypical phytases also exist [16–18]. Of the four typical phytases, BPPs are considered alkaline, whereas the others are classified as acid phytases (AP) based on their optimal pH levels. Furthermore, their hydrolysis products differ, with BPP producing Ins(2,4,6)P_3_ and AP generating Ins(2)P (Fig. 1). According to the initial dephosphorylation site, these phytases can also be divided into 3-phytase, 4-phytase, 5-phytase, and 6-phytase (Fig. 1). However, the sequential order of phytate dephosphorylation may vary across different species, even when they carry homologous phytases with identical structural bases [19–24].

**Table 1:** The biochemically or structurally characterized enzymes involved in InsP_6_ hydrolysis.

Clade	Representative sequence	Source of strain	Homologs^*^	Conserved, characteristic sequence motifs			
**β-Propeller Phytases (BPP)** [78]				**YG**	**EGXXXD**	**DXEG**	**DG**
ABL	ABL86758	*Bacillus subtilis* ARRMK33	AAC31775.1; AAC38573.1; AAM74021.1; ABP02074.1; ACZ57955.1; AEC13691.1; AFD29183.1; AVV65759.1; CAM58513.1; CRL66425.1; O31097; O66037; P42094; ABQ66899.1; CQR59166.1; MBO9525759.1	YG (159–160)	EGMAAD (211–216)	DIEG (258–261)	DG (314–315)
AQX	AQX17444	*Pseudomonas* sp. FB15	AAN55555.1; ADZ99372.1; ADZ99940.1; AEQ29499.1; AHM26864.1; AJF36073.1; UNF21334.1	YG (418–419)	EGCVAD (468–473)	DVEG (514–517)	DG (571–572)
ACJ	ACJ35482	*Pedobacter nyackensis* MJ11	-	MG (170–171)	ESIAVD (229–234)	DNEG (274–277)	DG (329–330)
QJA	QJA16365	*Arthrobotrys oligospora* ATCC 24927	-	YG (529–530)	EGCVVD (583–588)	DVEG (632–635)	DG (691–692)
**Histidine Acid Phytases (HAP)** [79]				**RHGXRXP**		**HD/HAE**	
AAA	AAA32705	*Aspergillus niger*	AAB96872.1; AAN10115.1; AAU93517.1; CAC48195.1; D4ANW6; O00085; O00093; O00100; O00107; P34753; Q0CLV1; Q9C1T1	RHGARYP (81–87)		HD (361–362)	
ABG	ABG88857	*Aspergillus niger* strain WP1	2GFI_A (PDB); AAA02934.1; ABU49229.1; ADF49635.1; P34755; ABN04184.1	RHGERYP (65–71)		HD (321–322)	
050	WP_057099050	*Bacteroides thetaiotaomicron*	-	RHGARFP (58–64)		HAE (323–325)	
ACJ	ACJ51391	*Bifidobacterium longum*	-	RHGSRGL (74–80)		HAE (429–431)	
797	WP_027265797	*Legionella pneumophila*	-	RHGDRTP (33–39)		HD (280–281)	
896	WP_198495896	Metagenome samples	JGI1357J11328_100303543^*^	RHGNRTP (45–51)		HD (293–294)	
AAM	AAM23271	*Klebsiella* sp.strain ASR1	AAL59319.1; ABW76125.1	RHGIRPP (40–46)		HD (306–307)	
AAR	AAR89622	*Citrobacter freundii*	AAR87658.1; AAS45884.1; ABI95370.1; ABI98040.1; ABX75421.1; ABX80238.1;ACB54699.1; AFG25721.1; BAQ94585.1; QPN96261.1; P07102; WP_117343249.1; P19926; AIE90144.1	RHGVRAP (38–44)		HD (324–325)	
**Cysteine Phytases (CP)** [80]				**DH (WPD loop)**		**HCXXGXGRT (P-loop)**	
AAQ	AAQ13669	*Selenomonas ruminantium* JY35	ABA18187.1; ABC69358.4; ABC69359.4; ABC69361.2; ABC69367.2; ADG23212.1; ADG23213.1; CAE79111.1; WP_011590872.1	DH (223–224)		HCEAGVGRT (251–259)	
AWN	AWN00236	*Streptacidiphilus melanogenes*	-	YF (240–241)		HCTAGKDRT (268–276)	
**Purple Acid Phytases (PAP)** [15]				**GDXG**	**GNH[D/E]**	**VXXXH**	**GHXH**
ACR	ACR23329	*Triticum aestivum*	ANG56539.1; ACR23326.1; ACR23333.1; ACR23335.1; AEG77017.1; ACR23327.1; ACR23331.1; AEG77016.1; ADG07931.1; AEA39182.1; ACR23328.1; ACR23332.1; ABP96799.1; AAX71115.1; AAN74650.1; ACD87745.1; ADM32493.1; ADM88044.1; ADI50286.1; AAK49438.1; AAE83899.1; AAX20028.1; AJK28063.1; WP_017329228.1	GDLG (193–196)	GNHE (277–280)	VAGWH (356–360)	GHVH (396–399)
XP	XP_004504591	*Cicer arietnum L.*	NP_001242830.2	GDWG (52–55)	GNHD (123–126)	VVGHH (215–219)	GHDH (252–255)

^^*^^Homologous sequences from each clade showing >28% sequence identity are grouped together. All sequences included in the table are experimentally validated phytases. Sequences were retrieved from the National Center for Biotechnology Information (NCBI) database or the Protein Data Bank (PDB) database. Corresponding amino acid positions of the conserved sequence motifs are indicated in bracket.

InsPs constitute a significant portion of the DOP in coastal waters [10, 25] which is believed to originate from terrigenous inputs, including plant tissues and animal feces [7, 10, 26–28]. However, it remains unclear whether InsPs are produced in significant quantities in the ocean, despite marine macrophytes and phytoplankton being capable of producing InsPs, particularly phytate [29, 30]. Current research has primarily focused on the metabolic mechanisms and potential applications of phytate and phytases in human nutrition [31, 32], agricultural production [33–35] and commercial use [36, 37], whereas investigations into InsPs hydrolysis by marine microbiome remain limited. It has been reported that marine phytoplankton, including the coccolithophore *Emiliania huxleyi* [38] and the diatom *Phaeodactylum tricornutum* [27] can efficiently utilize phytate to support algal growth. Additionally, sediment microbiota in seagrass beds [30, 39] and mangroves [40] can degrade phytate through phytases. Metagenomic analysis has also revealed a widespread distribution of bacterial BPP in both coastal and open oceans [41]. Thus, the enzymatic degradation of phytate by marine microorganisms may provide them with a competitive advantage in acquiring phosphorous [42].

As a significant component of DOP in the ocean, InsPs hydrolysis may play a crucial role in microbial adaptation to phosphorus limitation, particularly in oligotrophic ocean areas. To address the knowledge gap regarding the marine source of InsPs and their potential contribution to the oceanic phosphorus cycle, we determined InsPs concentrations in marine macrophytes. Meta-omics analyses of samples from both pelagic and benthic marine environments, combined with enzymatic activity assays at the protein and community levels, were conducted to assess the feasibility of InsPs hydrolysis in natural environment. Overall, our study highlights the often-overlooked role of microbial InsPs hydrolysis in alleviating phosphorus limitation in the ocean, thereby contributing to a more comprehensive understanding of the global marine biogeochemical cycling of phosphorus.

## Materials and methods

### Sample collection

The natural seawater, sediments, the seaweeds (*Sargassum thunbergii*, *Gelidium amansii*, and *Enteromorpha prolifera*), and the seagrass (*Zostera marina*) were collected from the intertidal zone of Qingdao, China (36.06°N, 120.31°E). The seagrass samples of *Cymodocea rotundata*, *Enhalus acoroides*, and *Thalassia hemprichii* were collected from Li-An lagoon of Hainan, China (18.41°N, 110.05°E). The seaweed samples of *Macrocystis pyrifera*, *Saccharina japonica*, and *Caulerpa lentillifera* were purchased from local aquaculture companies (Shandong, China), where they were commercially cultivated in coastal regions.

### Extraction of InsPs from seawater, seaweeds, and seagrasses

Collected natural seawater was filtered through a 0.22 μm pore size Millex-GP filter (Millipore) to remove large organisms and solid particles. InsPs in the seawater samples were purified via titanium dioxide (TiO_2_) beads (Titansphere TiO 5 μm; GL Sciences, Japan), following a previous procedure [28] with some modifications. Briefly, 20 mg TiO_2_ beads were pretreated by washing once in deionized water, followed by one wash with 1 M perchloric acid (PA; Sinopharm, China), and then resuspended in 50 μl of 1 M cold PA. One ml of 11.6 M cold PA was added to 10 ml of seawater and the samples were left rotating at 4°C for 2 hours. The pretreated beads were then added to the resulting supernatant and rotated at 4°C for 2 hours. Beads were pelleted and washed twice in 1 M cold PA and then eluted by washing with 200 ml of cold 10% ammonium hydroxide (pH 10.0; Sinopharm, China) twice. The supernatants were then vacuum evaporated to a final volume of 50 μl for InsPs detection.

Biological samples were first freeze-dried and ground to a fine power, passing through an 80-mesh sieve, prior to the extraction of InsPs. 100 mg freeze-dried powder of each sample was extracted under mechanical agitation with 4 ml 0.66 M HCl for 2 hours at room temperature. The extract was centrifuged at 13 000 rpm, 4°C for 10 min. The resulting supernatant was concentrated to dryness under vacuum (30°C), re-suspended in 3 ml of 25 mM HCl, and subsequently centrifuged at 13 000 rpm, for 10 min at 4°C. InsPs were isolated from the resulting supernatant and concentrated using a modified ion-exchange chromatography procedure [43]. Briefly, the supernatant was carefully collected and applied to a strong anion-exchange (SAX) column (quaternary amine bonded silica, 100 mg/1 ml; Thermo Fisher Scientific, USA). The loaded SAX column was washed with 1 ml of 25 mM HCl. Resin-bound InsPs were then eluted using two 0.6 ml portions of 2 M HCl. The eluent was evaporated to dryness (30°C) and dissolved in 200 μl of ddH_2_O for InsPs detection. For comparison, *Sesamum indicum* seeds were used as a positive control; a total of 100 mg of freeze-dried seeds were first de-oiled with 1.5 ml of n-hexane at 25°C for 1 hour, followed by the extraction procedures described above. No orthophosphate was detected in these samples using the molybdate assay described below.

### Determination of InsPs concentration

To determine the presence of InsP_6_ in extracted samples, HPLC–MS analysis was performed using the Thermo Scientific Ultimate 3000 high performance liquid chromatography (HPLC) coupled with a high-resolution hybrid quadrupole time-of-flight (qTOF) mass spectrometer (Bruker impact HD, Germany). 10 μl samples were injected. A C18 reverse-phase column (SunFire C18 Column, 5 μm, 4.6 mm × 250 mm; Waters, USA) was used, with a column temperature of 30°C. The mobile phase consisted of 20 mM ammonium acetate, and 5% (v/v) methanol in Milli-Q water (pH 4.5) at a flow rate of 0.5 ml min^−1^. IP6 was detected in the negative mode by electrospray ionization (ESI). The MS ion source conditions were set as: spray voltage, 4 kV; end plate offset, 500 V; capillary temperature, 200°C; dry gases (nitrogen) flow rate, 6 l min^−1^; nebulization pressure, 0.5 bar. During operation, the mass analyzer operated in MS full-scan and Auto MS/MS scan mode. Data acquisition of mass spectra was controlled by inbuilt software (otofControl Version 3.4), and data analysis were performed by Compass DataAnalysis Version 4.2 (Bruker, Germany).

To quantify phytate in the microcosm studies it was detected by a spectrometric method using FeCl_3_ and sulfosalicylic acid as the coloring reagents [44]. To prepare the coloring reagents, 0.5 g of FeCl_3_ and 5 g of sulfosalicylic acid were dissolved in double distilled water and adjusted to a final volume of 50 ml. The reagent was diluted five times before use. A reaction mixture consisting of 100 μl of sample solution and 100 μl of diluted coloring reagent solution was then incubated at room temperature for 20 min, followed by the measurement of absorption at a wavelength of 500 nm.

### Bioinformatics

To build a local reference sequence database, the full-length amino acid sequences of functionally ratified enzymes (Table 1) involved in hydrolysis of InsPs were compiled (until Apr 26^th^, 2024) and retrieved from the National Center for Biotechnology Information (NCBI, https://www.ncbi.nlm.nih.gov/) database. The sequences with a similarity >28% were considered homologs of the same protein, and the best hit was selected as the representative sequence among other homologs (Table 1). All representative sequences were aligned via ClustalW [45] and sequence identities were determined using BioEdit 7.0 [46]. For comparison, the full-length amino acid sequences of alkaline phosphatases PafA [47], Psip1 [48], PhoA [49], PhoD [50], PhoK [51], and PhoX [52] (Table S1) were also retrieved from NCBI database for homology searching.

To determine the geographical traits of InsPs metabolism in marine environments, 60 polar seawater metagenomic samples [53] (NCBI BioProject accession no. PRJNA588686), 173 non-polar seawater metagenomic samples from the Tara Oceans project [54] (http://www.pangaea.de/), 76 coastal water samples, 34 coastal sediment samples, 35 deep sea sediment metagenomics samples, as well as 145 metatranscriptomic samples (105 seawater samples and 40 sediment samples) from the IMG/M database [55] (https://img.jgi.doe.gov/cgi-bin/m/main.cgi) were collected for analyses (Fig. S1).

### Sequence homology search and screening

Hidden Markov Model (HMM) profiles were created via the hmmbuild program from HMMER version 3.3.2 [56] (http://hmmer.org), using protein sequences that are either biochemically or structurally characterized (Table 1). Their homologs from metagenomes and metatranscriptomes were then obtained using the hmmsearch program to build a custom-made dataset with an initial e-value cutoff of 1E-30. To investigate the conservation of retrieved environmental sequences, the potential homologs of each enzyme were aligned by MUSCLE [57]. The key conserved residues involved in catalysis and substrate binding were collected (Table 1) and utilized to screen the qualified environmental sequences. For sequences lacking validated structures, phylogenetic trees of their homologs were constructed using FastTree [58], and visualized with EvolView [59]. Unqualified sequences were excluded from the homologs dataset. The relative abundance of each gene was normalized against the average abundance of the 10 universal bacterial marker genes in each metagenome/metatranscriptome [60].

### Experimental validation of environmental phytase sequences

Each environmental sequence of phytases (Table S2) was chemically synthesized (BGI, China), and cloned into the pET28a vector (Novagen, China) with a N-terminal His tag, and then transformed into *Escherichia coli* strain C43 (DE3) [61]. The signal peptide of each sequence was predicted by SignalP 6.0 [62] and removed before chemical synthesis. Recombinant *E. coli* cells were cultured at 37°C in Lysogeny Broth medium to an OD_600_ of 0.8 ~ 1.0, and then induced at 16°C for 18 hours with 0.3 mM isopropyl β-d-1-thiogalactopyranoside. Cells were then lysed by a high-pressure French press, and the supernatants containing the protein of interest were prepared from the disrupted cells by centrifugation, and the recombinant proteins were purified with Ni^2+^-NTA resin (QIAGEN, Germany). The protein concentration was determined using the bicinchoninic acid assay [63]. The phytase activity was assessed by quantifying the release of orthophosphate using ammonium molybdate as the chromogenic reagent [64]. A standard assay mixture consisted of 50 μl prepared enzyme and 100 μl of solution buffer, with the final concentration of 2.5 mM sodium phytate, 10 mM CaCl_2_, and either 100 mM Tris-malate buffer (pH 8.0) or 100 mM acetic acid–sodium acetate buffer (pH 4.5). The standard assay mixture was incubated at 25°C for 30 min and then stopped by adding 150 μl of trichloroacetic acid (10% w/v). The released orthophosphate in the mixture was analyzed by adding 150 μl of coloring reagent containing 1% (w/v) ammonium molybdate, 3.2% (v/v) sulfuric acid, and 7.2% (w/v) ferrous sulfate. The mixture was then centrifuged at 13 000 rpm, and 200 μl of the resulting supernatant was utilized for measuring the absorbance of orthophosphate at a wavelength of 700 nm.

### Kinetic assay of environmental phytases

The two phytases, BPP_ABL, and PAP_XP, with the highest relative abundance in metagenomes and metatranscriptomes respectively, were selected for kinetic analysis. To determine the optimal temperature for phytase activity, reaction mixtures were incubated at 10, 20, 30, 40, 50, and 60°C. The optimum pH was examined using the following buffer solutions: sodium acetate, pH 4.5–5.5; Tris-maleate, pH 5.5–7.0; Tris–HCl, pH 7.0–8.0; The optimum CaCl_2_ concentrations of BPP_ABL and PAP_XP were examined using Tris–HCl buffer (pH 7.0) and Tris-maleate buffer (pH 6.5) at values of 0, 1.25, 2.5, 3.75 and 5.0, respectively. After the optimal pH, temperature and CaCl_2_ concentration had been determined, all measurements were performed under these optimal conditions. To determine the kinetic parameters, 13.10 μg and 7.24 μg enzymes of BPP_ABL and PAP_XP were added to sequentially diluted solutions of InsP_6_ with different concentrations in 200 μl of 0.1 M solution buffer under the condition of pH 7.0 (Tris–HCl buffer), 40°C, 1.25 mM CaCl_2_ and pH 6.5 (Tris-maleate buffer), 50°C, 2.5 mM CaCl_2_, respectively. After an incubation period of 30 min, the liberated phosphate was quantified using the aforementioned ammonium molybdate method [64]. The activity was expressed as micromoles of phosphate liberated per minute. The kinetic constants (*K*_m_ and *v*_max_) were calculated from the Lineweaver-Burk plots of the data. For the calculation of *k*_cat_, the following molecular masses were used: BPP_ABL, 37 kDa; PAP_XP, 35 kDa.

### Detection of phytate degradation ability of environmental samples

To evaluate the InsPs degradation capacity of various intertidal samples, a total of 500 ml seawater sample, 124.5 g of sediment sample, and 24.15 g of seagrass sample were collected from multiple sampling sites in the Qingdao intertidal zone (36.06°N, 120.31°E). Microorganisms in the seawater sample were directly filtered onto a 0.22 μm pore size Millex-GP filter. The sediment sample was suspended in sterilized 35‰ artificial seawater (Sigma, USA), and microorganisms in the supernatant were then filtered onto a 0.22 μm pore size Millex-GP filter. For the seagrass samples, the phyllospheric microorganisms were scraped using sterile forceps, and then filtered onto a 0.22 μm pore size Millex-GP filter. The acquired different filters were evenly divided into several parts. Half of the filter parts (three repeats of each sample) were separately cultivated in 20 ml sterilized 35‰ artificial seawater supplied with phytate with a final concentration of ~2 mM (pH 8.0). The incubation was conducted at 25°C for 3 days. Artificial seawater with added phytate but without biological samples was used as a control. The residual phytate of each day was detected by a spectrometric method using FeCl_3_ and sulfosalicylic acid as the coloring reagents [44] as aforementioned.

The microbes on the rest half of the filter parts (three repeats of each sample) were eluted with sterile glass beads. The eluted microbial solutions were gradient diluted (10^0^ ~ 10^−3^) and then spread onto solid culture media plates with 4 mM phytate as the sole P source, and then incubated at 25°C until transparent circles appeared.

To access the genetic potential of phytases, metagenomic DNA from 1 L seawater-derived filter, 5 mg of sediment and 5 mg of seagrass phyllospheric attachment were extracted using the E.Z.N.A. Soil DNA Kit (Omega, USA). Real-time fluorescent quantitative PCR (RT-qPCR) was conducted on a Light Cycler II 480 System (Roche, Switzerland). The PCR mixture contained 10 μl TB Green Premix Ex Taq II (TaKaRa, Japan), two pmole forward and reverse primers (Table S3), 50 ng DNA, and nuclease-free water to reach a final volume of 20 μl per well. The thermal cycling consisted of an initial denaturation at 95°C for 30 s followed by 50 cycles of 95°C for 5 s and 65°C for 30 s. The V6 invariant region of 16S rRNA genes (Table S3) were used as a reference to normalize the gene abundances of phytases [65]. The genetic abundances were determined via the ratios of Cp values between the 16S rRNA gene and phytase genes (Cp_REF_/Cp_TARGET_). The qPCR primers of phytases were designed by Beacon Designer 8 (www.premierbiosoft.com, Table S3).

### Statistical analysis and visualization

To determine the taxonomic assignment of environmental sequences, homologs were aligned against the non-redundant protein sequences database using BLASTP [66]. The best hit of each homolog was retrieved, and its taxon was recorded. Data cleaning and processing of raw datasets derived from sequence searching and phylogenetic analysis were performed via scripts compiled in Python code (https://www.python.org/). The processed data were analyzed and visualized using the R software packages [67]. Briefly, relative abundances and phylogenetic diversities of phytate-related genes were visualized using the “ggplot2” package [68]. Venn diagrams were created based on the taxonomic compositions of phytate-related microbiota via the “ggVennDiagram” package [69]. Principal coordinates analysis, transformation-based redundancy analysis, alpha-diversity analysis as well as distance-decay analysis were performed using the “vegan” package [70] and visualized by “ggplot2”package [68]. *P* values among different samples were calculated via the “ggsignif” package (https://CRAN.R-project.org/package=ggsignif). The Sankey diagrams and Circos plots of the taxonomic profiling were generated using the “ggalluvial” package [71] and “circlize” package [72], respectively. To reveal the mechanisms governing the assembly of InsPs degradation traits, normalized stochasticity ratio (*NST*) was applied to characterize the ratio of stochasticity to determinism via “NST” package [73] with 50% as the boundary point. The geographical location of meta-omics samples was constructed by Ocean Data View [74]. The phylogenetic trees of validated phytases were constructed by MEGA 7.0 [75] using the Neighbor-joining method and the Maximum Likelihood method respectively, with a bootstrap value of 1000. Graphic layouts were adjusted via Adobe Illustrator CS5.

## Results

### Concentrations of InsPs in marine macrophytes and coastal seawaters

**Figure 2:**
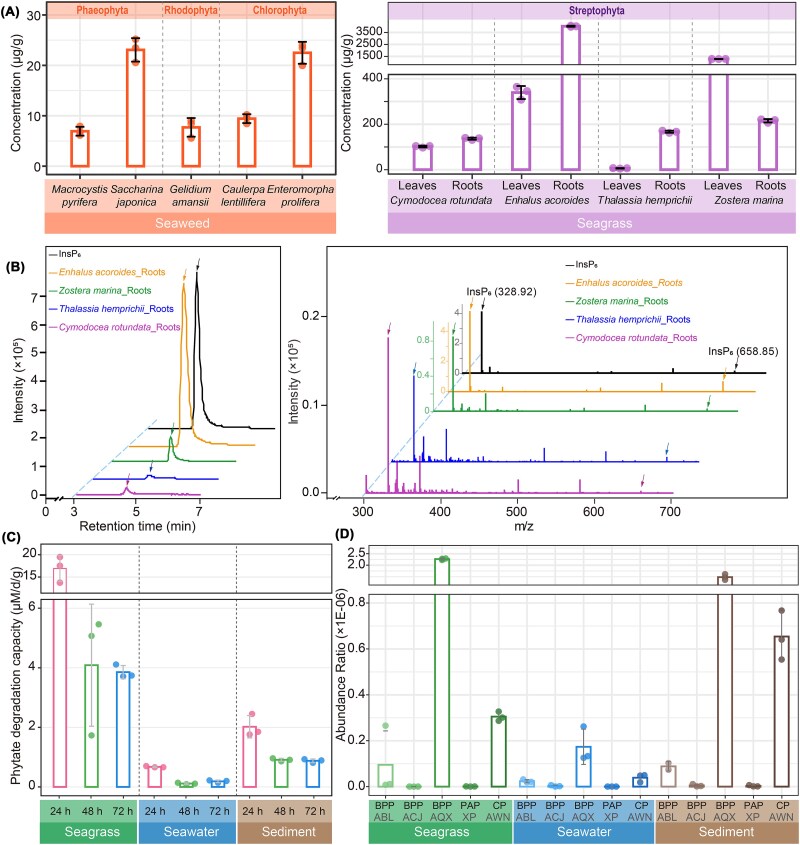
Concentration of inositol phosphates in biological samples and phytate hydrolysis activity by coastal marine microbiota. (**A**) concentrations of inositol phosphates (InsPs) measured in various marine samples, including seaweed and seagrass, expressed per gram of dry weight. (**B**) HPLC-MS analysis of InsPs (inositol phosphates) in seagrasses. InsPs were extracted from the roots of four seagrass species—*C. rotundata, E. acoroides, T. hemprichii*, and *Z. marina*—and analyzed using HPLC-MS in the negative electrospray ionization (ESI) mode. A prominent InsPs peak was observed at ~5 minutes, co-eluting with the InsP_6_ standard (left panel). The intact mass spectrometry (MS) scan of InsPs from these samples exhibited a similar spectral pattern to InsP_6_, with singly charged species at m/z 658.85 and doubly charged species at m/z 328.92 (right panel). (**C**) Phytate degradation capabilities of coastal microbiota from the seagrass phyllosphere, seawater, and sediment off the coast of Qingdao, China. InsPs degradation is evaluated based by measuring the rate of phytate consumption, expressed in μM per day per gram of sample. (**D**) Quantification of phytase gene abundance using qPCR, reported as gene copies per gram of sample. BPP, β-propeller phytases, represented by three distinct clades (ABL, ACJ, AQX); PAP, purple acid phytases represented by the XP clade; CP, cysteine phytases represented by the AWN clade.

To investigate the origins of InsPs in costal marine ecosystems, we collected various seaweed and seagrass species (Fig. 2A) to determine InsPs concentrations using a colorimetric assay involving FeCl_3_ and sulfosalicylic acid [44]. The samples included three categories of seaweeds, namely brown algae (Phaeophyta), red algae (Rhodophyta), and green algae (Chlorophyta), along with four types of seagrasses and seawater from above the seagrass beds. InsPs in various types of seaweeds exhibited divergent concentrations, generally below 30 μg/g dry weight (Fig. 2A). InsPs levels in seagrasses were one to two orders of magnitude higher than those in seaweeds. Typically, concentrations in roots were several times higher than in leaves, suggesting that roots are likely the primary accumulators of InsPs. Indeed, the InsPs content in *E. acoroides* roots and *Z. marina* roots were 4019.88 ± 47.10 μg/g and 215.23 ± 7.26 μg/g, respectively. InsPs concentrations in seawater samples from seagrass beds reached hundreds of nanomolar (196.29 ± 2.83 nM), an order of magnitude higher than in typical coastal seawater (38.62 ± 8.34 nM).

To identify the major InsPs species in these samples and validate the effectiveness of the aforementioned assay in quantifying InsPs, we also analyzed seagrass root extracts using HPLC–MS (Fig. 2B). The extracted InsPs from these root samples were eluted as a major peak at ~5 min, coinciding with the InsP_6_ standard (Fig. 2B, left panel). A mass spectrometry scan of the intact ion in negative ESI mode revealed the presence of both singly and doubly charged InsP_6_ species, detected as [M-H]^−^ at m/z 658.85 and [M-2H]^2−^ at m/z 328.92 respectively [76]. Thus, seagrass roots appear as a significant source of InsPs, particularly InsP_6_. Overall, our findings suggest that the ocean may serve as a substantial reservoir of InsPs, with marine macrophytes, including seaweeds and seagrasses, acting as significant contributors alongside running offs of InsPs originated from terrestrial plants.

### InsPs degradation by coastal marine microbiota

To investigate the degradation of InsPs by microbial communities in coastal marine environments, samples were collected from the seagrass phyllosphere, seawater and sediment in the coastal ocean of Qingdao, China. Among these, the seagrass phyllosphere microbiota demonstrated the highest InsPs degradation activity, with a rapid decrease of phytate concentrations observed within 24 hours (Fig. 2C, Fig. S2). To further assess potential involvement of phytases in the InsPs degradation, quantitative PCR was employed to measure the abundance of three major phytases in these samples. Results indicated that the AQX clade of BPP phytases and the AWN clade of CP phytases were significantly more abundant than the others (Fig. 2D), highlighting their key roles in phytate degradation in these samples. Overall, these findings provide compelling evidence that microbial-driven InsPs degradation is a prevalent process in costal marine ecosystems.

### Relative abundance of key genes involved in global marine InsPs hydrolysis

**Figure 3:**
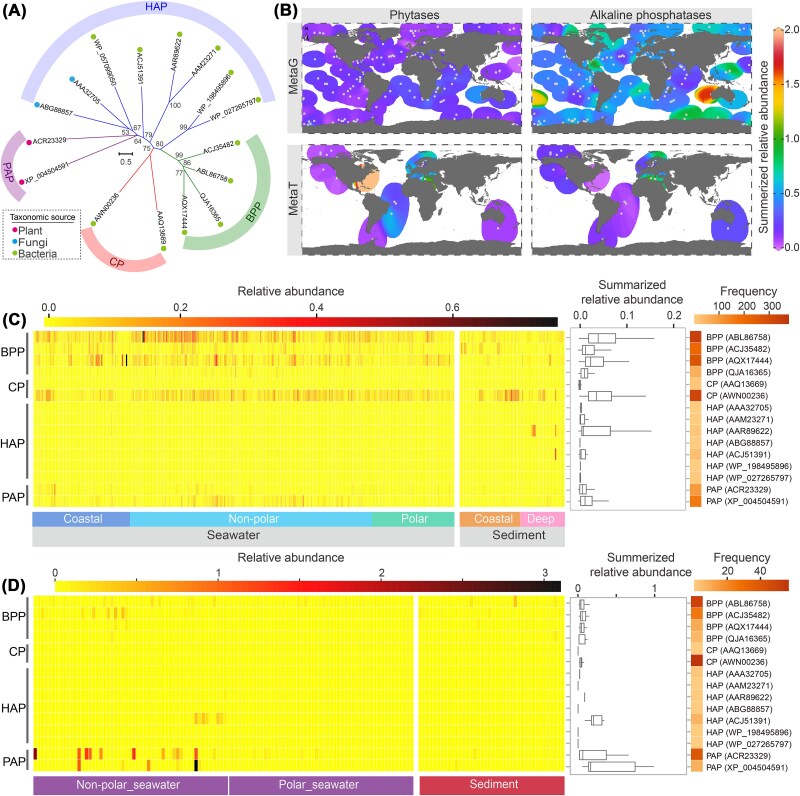
Relative abundance of key phytase genes in marine metagenomic and metatranscriptomic samples. (**A**) A phylogenetic tree showing four primary classes of phytases, constructed from representative enzymes selected based on sequence similarities >28% in each clade. Each leaf in the tree is labeled with the NCBI accession number of its representative enzyme, with colorful dots preceding the leaf names to indicate the taxonomic distribution of each enzymes. (**B**) Geographic distribution and relative abundance of phytases and alkaline phosphatases in the metagenomic and metatranscriptomic samples analyzed in this study. MetaG, metagenomes; MetaT, metatranscriptomes. Background coloring is based on an interpolation between sampling points shown in dark gray. (**C, D**) The relative abundances and prevalence of genetic potential of phytases from (**C**) metagenomic samples and (**D**) metatranscriptomic samples. The relative abundances of phytase and alkaline phosphatase genes were normalized to 1 via the average abundance of 10 marker genes. Box plots summarize the relative abundances of each protein across all metabiomes, while a heatmap on the right side displays the frequency of samples in which genetic potentials were detected.

To elucidate the distribution of genetic potentials related to InsPs metabolism in marine environments, we collected over 100 functionally validated phytase sequences. These sequences represent genetically, biochemically or structurally characterized *bona fide* phytases which were grouped into clades based on catalytically relevant motifs, resulting in four distinct clades of BPPs, eight clades of HAPs and two clades of PAPs and CPs (Table 1, Fig. 3A). Using these well-defined clades as references, we analyzed 381 metagenomes and 145 metatranscriptomes from pelagic and benthic oceanic environments (Fig. S1) using HMM based hmmsearch using a conserved e-value cutoff of 1E-30. For comparison, the relative abundance of alkaline phosphatases, which are essential enzymes involved in DOP degradation and phosphate release [77], was also investigated (Table S1). After filtering sequences based on their conservation at key functional sites [15, 49–52, 78–80] (Table 1, Fig. S3, S4), we identified 6466 candidates of phytases, including 6108 from metagenomes and 358 from metatranscriptomes (Table S4), and 21 291 candidates of alkaline phosphatases, including 20 504 from metagenomes and 787 from metatranscriptomes (Table S5).

We then calculated the relative abundance of these genes in the omics data by normalizing to the average abundance of universal bacterial marker genes [81] and assessed their geographical distributions (Fig. 3B). The candidates for both phytases and alkaline phosphatases demonstrated a wide distribution across metagenomic samples. As a critical indicator of high phosphorus depletion and stress [77], alkaline phosphatases were approximately one order of magnitude higher in relative abundance than phytases (Fig. 3B, Fig. S5), which aligns with its versatile role in facilitating the assimilation of diverse DOP compounds in the ocean [82]. In contrast to metagenomic data, phytases displayed more pronounced transcriptional activity compared to alkaline phosphatases, as evidenced by the greater abundance of transcripts in certain metatranscriptomic samples from the Atlantic Ocean. This observation points to a recurring presence of InsPs, particularly transient InsP_6_ pulses, underscoring the role of InsPs hydrolysis as an active and essential pathway for phosphate release in marine environments. Of the various phytase types, the alkaline phytase BPP exhibited the highest prevalence across metagenomes in comparison to the acid phytases (Fig. S6). Among the acid phytases, the CP clade represented by the AWN00236 sequence was among the most prevalent, whereas HAPs were rare (Fig. 3C). The PAP clades, although broadly distributed, showed low abundance in metagenomes; however, PAP transcripts were more abundant in metatranscriptomes than those of the other three phytases, particularly in non-polar seawater samples (Fig. 3D). This distribution suggests an active role for PAPs in the marine environments.

### Taxonomic assignment of the microbial community involved in global marine InsPs hydrolysis

**Figure 4:**
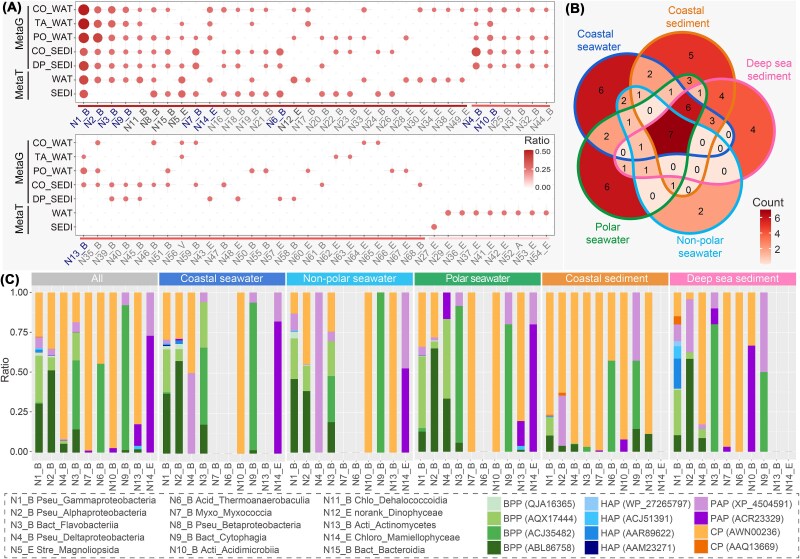
The taxonomic composition of microbial communities associated with phytate hydrolysis. (**A**) The taxonomic composition of microbiota in different metagenomic and metatranscriptomic sample types at class level. The total frequencies of classes in each sample type were normalized to 1. The top 15 most abundant classes are highlighted in grey. The top 10 classes in metagenomic samples are indicated in blue. B, bacteria; E, Eukaryota; a, archaea; V, viruses. CO_WAT, coastal seawater; TA_WAT, non-polar seawater; PO_WAT, polar seawater; CO_SEDI, coastal sediment; DP_SEDI, deep sea sediment; WAT, seawater; SEDI, sediment. (**B**) The Venn diagram of taxonomic composition at class level in metagenomic samples. The intersections exhibited the quantities of taxonomic classes shared among five sample types. (**C**) The taxonomic composition of representative phytases in the top 10 most abundant classes from metagenomic samples. The total frequency of each class was normalized to 1. The NCBI accession numbers enclosed in the brackets were utilized to indicate the representative enzymes. Pseu, Pseudomonadota; Bact, Bacteroidota; Stre, Streptophyta; Acid, Acidobacteriota; Myxo, Myxococcota; Acti, Actinomycetota; Chlo, Chloroflexota; Chloro, Chlorophyta.

We investigated the identity and diversity of marine microbes encoding these InsPs hydrolysis enzymes. Taxonomic profiling of the retained environmental sequences from omics samples identified at least 99 classes associated with InsPs degradation, including 39 named classes from the Bacteria, 27 from Eukaryota, one from Archaea, and one from Viruses (Fig. 4A, Table S6). A total of 28 named classes were co-found in both metagenomics and metatranscriptomic samples, and the majority of named classes (31 out of 68) were solely detected from metagenomes (Fig. 4A). Among the 59 named classes from metagenomic samples, γ- and α-proteobacteria from Pseudomonadota were the most abundant (Fig. 4A). Of the named classes, seven were found across all five mategemomic sampling environments and belonged exclusively to the Bacteria kingdom. Four bacterial classes were unique to sediment samples, whereas a single class, Mamiellophyceae from the phylum Chlorophyta, was present in all three seawater samples (Fig. 4B). Unique classes observed within each sampling environment accounted for ~57% (23 out of 59) of the named classes, with marine seawaters hosting the largest share (14 out of 23).

Indeed, the top 10 classes from metagenomic samples accounted for more than 55% of the total taxonomic abundance in metagenomic sediment samples (65.8% in coastal sediments and 55.1% in deep-sea sediments) whereas they exceeded 77% in metagenomic seawater samples (77.5% in coastal seawaters, 81.9% in non-polar seawaters, and 86.7% in polar seawaters), demonstrating their predominate role in facilitating InsPs hydrolysis in marine environments. Seven of 10 top classes were consistently present in all five types of metagenomic samples, with the exception of Thermoanaerobaculia from Acidobacteriota, Myxococcia from Myxococcota, and Mamiellophyceae from Chlorophyta, among which Thermoanaerobaculia was exclusively detected in sediment environments. Moreover, the composition patterns of phytase homologs within each class displayed distinct variations across different sampling environments (Fig. 4C). The majority of members from the top two classes (i.e. γ- and α-proteobacteria), carried orthologues of BPPs. In contrast, the Flavobacteriia class from Bacteroidota exhibited a greater diversity of phytases, including genetic potentials from the BPP, CP, and PAP families (Fig. S7). Exceeding 90% of members from Deltaproteobacteria (91.6%), Myxococcia (98.7%), and Acidimicrobiia (97.2%) carried homologs of the AWN00236 clade from the CP family. Meanwhile, the wide phylogenetic distribution of AWN00236 across marine environments suggests its strong adaptability in different physicochemical conditions. The phylogenetic diversity of AWN00236 in seawater and sediment metatranscriptomic samples further supports this idea (Fig. S8).

### Biogeographical traits of marine phytate hydrolysis

**Figure 5:**
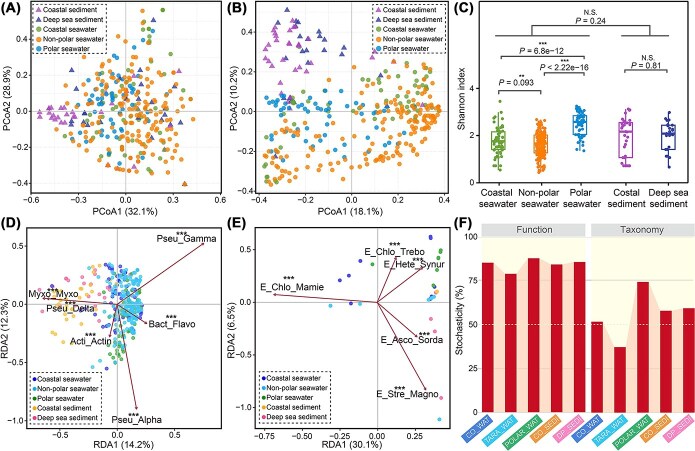
Biogeographic traits of phytate hydrolysis in marine environments. (**A–B**) Principal coordinates analysis of all metagenomic samples, illustrating biogeographic patterns based on Bray–Curtis dissimilarities derived from (**A**) abundances and (**B**) taxonomic compositions of genetic potentials associated with phytate hydrolysis. (**C**) Comparison of α-diversity among microbial species involved in phytate hydrolysis across the five metagenomic sample types. ^***^, *P* < .001; ^**^, *P* < .01; N.S. not significant. (**D, E**) Transformation-based redundancy analysis revealed (**D**) the critical bacterial classes and (**E**) non-bacterial phyla driven the phytate hydrolysis process. Pseu, Pseudomonadota; Myxo, Myxococcota; Bact, Bacteroidota; Acti, Actinomycetota; Gamma, Gammaproteobacteria; Alpha, Alphaproteobacteria; Delta, Deltaproteobacteria; Myxoc, Myxococcia; Flavo, Flavobacteriia; Actin, Actinomycetes; E, Eukaryota; Asco, Ascomycota; Chlo, Chlorophyta; Stre, Streptophyta; Hete, Heterokontophyta; Mamie, Mamiellophyceae; Synur, Synurophyceae; Magno, Magnoliopsida; Sorda, Sordariomycetes; Trebouxiophyceae, Trebo. The total genetic potential abundance and taxonomic composition of phytate hydrolysis-related enzymes in each metagenomic sample were normalized to 1, with the percentages of variation displayed on the axes. (**F**) Assembly processes of functional genes and microbial communities involved in marine phytate degradation, based on *NST* calculations, with 50% serving as the boundary between predominantly deterministic (<50%) and stochastic (>50%) assembly processes.

Based on the relative abundance of genetic potentials in metagenomes, degradation of InsPs in the oceans was primarily mediated by several key phytases from the BPPs (ABL86758, AQX17444, and ACJ35482) and CPs (AWN00236). No significant discrepancies were observed in the distribution patterns of these phytases across the five distinct marine environments (Fig. 5A), suggesting comparable functional potentials for InsPs degradation across different oceans, despite geographical variation. In contrast, the microbial communities responsible for InsPs hydrolysis in seawaters and marine sediments showed notable differences (Fig. 5B). Although species richness did not differ significantly between seawater and sediment samples, the compositional variation of microbial communities among seawater samples from different regions tended to be statistically significant (Fig. 5C). Further analysis revealed that the differences in the abundance distribution of co-occurring classes was the primary factor contributing to the dissimilarities between seawater and sediment communities. In general, γ-, α-proteobacteria, and Flavobacteriia were more dominant in seawaters, whereas sediments featured higher prevalence of δ-proteobacteria and Myxococcia (Fig. 5D). Although they had low relative abundance and limited spatial distribution, non-bacterial phyla, such as Chlorophyta and Ascomycota from Eukaryota remained integral components of the microbial community and contributed to marine InsPs degradation (Fig. 5E). Additionally, higher plants from the Streptophyta phylum were identified as InsPs decomposers in marine seawaters and sediments (Fig. 5E), carrying potential orthologues of PAPs.

To examine the mechanism underlying the assembly of microbial community and functional traits involved in InsPs hydrolysis, *NST*, a null model-based approach, was applied to assess the ecological stochasticity and determinism quantitatively [73]. The *NST* calculations revealed that the assembly of InsPs hydrolysis genes and associated microbial communities were highly stochastic (Fig. 5F). The stochastic strengths suggested that the assembly of functional genes in oceans was predominantly a stochastic process (average *NST* = 84.42%), indicating that ecological stochasticity, dispersal, or drift possibly may influence their distribution traits [83–85]. In contrast, the microbial communities involved in InsPs degradation displayed lower stochasticity (average *NST* = 56.14%), with even lower ratios in the non-polar oceans (*NST* = 37.27%). In these regions, deterministic processes such as environmental filtering and biological interactions [86–88], likely affect microbial fitness and shape community structure. Overall, considering the varied taxonomic compositions coupled with homogeneous functional traits (Fig. 5A, 5B), the assembly of functional microbial assemblage associated with marine InsPs hydrolysis aligns with neutral theory [83, 84, 89], emphasizing the role of stochastic processes in shaping ecological community structure. This framework is consistent with the nature of phytases as secretory proteins that facilitate the degradation of phytate under relatively similar extracellular conditions.

### Functional validation of environmental phytases

**Figure 6:**
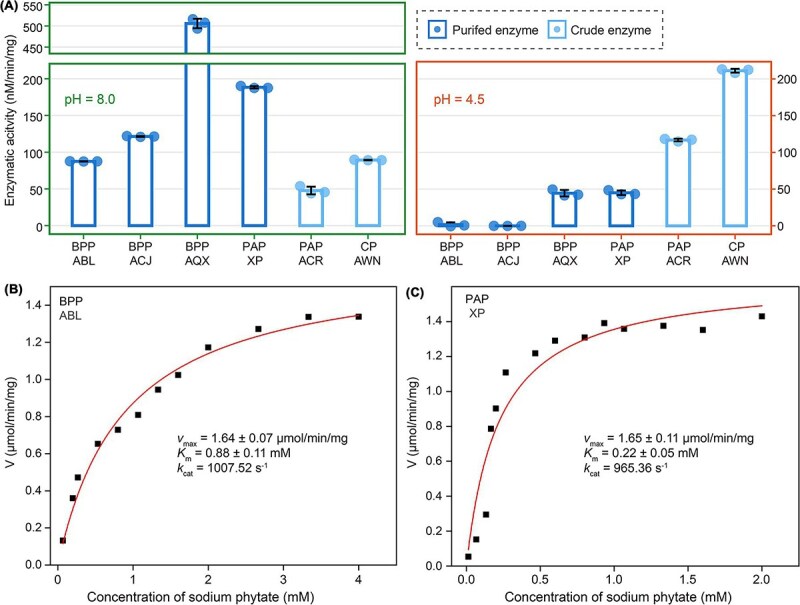
Phytate hydrolysis activities and of metagenomic-derived sequences. (**A**) The enzymatic activities of environmental phytase sequences at pH 8.0 and 4.5, respectively. The enzymatic hydrolysis capacity of environmental phytase per milligram was assessed by the production rates of phosphorus. (**B, C**) Enzymatic kinetic parameters for InsP_6_ dephosphorylation by (**B**) BPP_ABL and (**C**) PAP_XP. The enzymatic activities of BPP_ABL and PAP_XP were evaluated under their respective optimal conditions: 1.25 mM Ca^2+^, 40°C, pH 7.0 for BPP_ABL, and 2.5 mM Ca^2+^, 50°C, pH 6.5 for PAP_XP. The enzymatic activity rates (V) were expressed as micromoles of phosphate liberated per minute by per milligram of enzyme. ABL, ABL86758; ACJ, ACJ35482; AQX, AQX17444; XP, XP_004504591; ACR, ACR23329; AWN, AWN00236.

To validate the predicted functions of environmental sequences involved in InsPs degradation, we selected those with relatively high abundances, including three clades from BPPs (ABL86758, ACJ35482, and AQX17444), two from PAPs (XP_004504591 and ACR23329), and the AWN00236 clade from CPs. These sequences were chemically synthesized and heterologously expressed in recombinant *E. coli*. A diverse range of sampling oceans, sample types (seawaters or sediments) and phylogenetic distributions were considered to ensure comprehensive representation of the selected sequences (Fig. S9). These proteins were then purified and the predicted enzyme activities were assayed at either pH 4.5, optimal for acid phytases, or at pH 8.0, close to the typical pH of natural seawater. Based on the rates of inorganic phosphate release from InsPs, purified enzymes from BPPs exhibited significantly higher activity at pH 8.0 than at pH 4.5 (Fig. 6A), consistent with their characterization as alkaline phytases [12]. XP_004504591 from PAPs also showed higher activity under alkaline reaction conditions [15, 90] although the underlying mechanism remains unclear. Additionally, the phytate hydrolysis activities of crude enzymes from two other acidic phytases (ACR23329 from PAPs, and AWN00236 from CPs) were detectable at pH 8.0, albeit at lower levels compared to pH 4.5 (Fig. 6A). This suggests that the genetic potential of acidic phytases retrieved from meta-omics samples is capable of functioning effectively in alkaline marine environments.

To assess the catalytic efficiency of phytase derived from marine environments, ABL86758 from BPPs and XP_004504591 from PAPs, two phytases with the highest relative abundance in metagenomes and metatranscriptomes, respectively, were selected (Fig. 3). The enzymatic parameter detection was subsequently conducted under their respective optimal conditions (Fig. S10). The *K*_m_ (0.22 ± 0.05 mM) and *k*_cat_ (956.39 s^−1^) of the marine PAP XP_004504591 were comparable to those of the terrestrial PAPs reported previously, with *K*_m_ values ranging from 0.08 to 0.30 mM and *k*_cat_ values ranging from 523 to 908 s^−1^ [20, 91]. As for ABL86758 from BPPs, despite its moderate substrate affinity (*K*_m_ = 0.88 ± 0.11 mM) compared to terrestrial BPPs (0.087 ~ 4.70 mM), the marine BPP exhibited significantly higher catalytic efficiency (*k*_cat_ = 1007.52 s^−1^) relative to terrestrial ones (2.93 ~ 64 s^−1^) [12], which suggests the robust capability of BPPs for InsPs hydrolysis in marine environments. Moreover, microbial consortia capable of degrading phytate were also identified in the seagrass phyllosphere, seawater, and sediment (Fig. S11). Overall, these findings elucidate the function of phytase at both the enzymatic and microbiome levels, highlighting the unneglectable role of InsPs hydrolysis in the marine environment.

## Discussion

**Figure 7:**
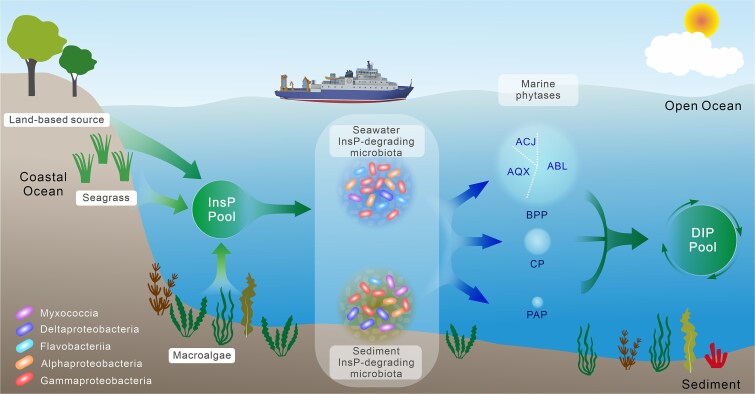
The schematic diagram illustrates the process of InsP hydrolysis in marine environments, integrating the relative abundance and taxonomic composition of potential phytase genes. Production of dissolved inorganic phosphorus though InsP degradation by phytases. Arrowheads indicate the directional flow of InsPs, including their sources and potential sinks. The major microbial contributors to InsPs hydrolysis are highlighted. Circles in the diagram denote the total relative abundance of potential phytase genes in different sampling environments. The size of each circle reflects the relative abundance of phytases, emphasizing variations in the hydrolysis potential across different habitats.

In contrast to other DOPs, InsPs are unique in their ability to provide phosphorus during early life stages, such as seed germination [92, 93]. Consequently, InsPs have been extensively studied in the context of phosphorus storage and cell signaling. However, their role in oceanic phosphorus cycling remains largely unexplored. Our findings (Fig. 7) indicate that marine macrophytes, including seagrasses and macroalgae, may contribute significantly to oceanic InsPs. Seagrasses, among the most productive ecosystems widely distributed along temperate and tropical coastlines, exhibit relatively high InsPs levels. Observed concentrations in seawater surrounding seagrass meadows reach hundreds of nanomolar [94, 95]. Despite covering less than 0.2% of the world’s ocean area, seagrass meadows store up to 19.9 Pg organic carbon [96] and contribute ~10% of oceans’ annual carbon burial [97]. Therefore, InsPs derived from seagrasses are predicted to constitute a considerable source of coastal DOP.

Similarly, significant concentrations of InsPs were detected in macroalgae from the Phaeophyta, Rhodophyta, and Chlorophyta groups. Macroalgae, with a global carbon sequestration potential of ~173 Tg annually, are likely crucial contributors to pelagic InsPs [98]. These InsPs are transported across the open and deep ocean as well as marine sediment [98–100] via lateral advection and sinking [98, 101]. Moreover, cosmopolitan marine phytoplankton, such as *E. huxleyi* [38] and *P. tricornutum* [27], have demonstrated the ability to utilize phytate as a phosphorus source, further underscoring the widespread presence of phytate in the global oceans. Nevertheless, challenges persist in distinguishing between various forms of InsPs and collection sufficient field data from open oceans. Therefore, large-scale determinations of marine InsPs stoichiometry are essential to fully elucidate their role in global phosphorus cycling.

Phytases alone are incapable of fully hydrolyzing all phosphate groups from InsPs (Fig. 1), and complete dephosphorylation is often facilitated by additional enzymes such as acid or alkaline phosphatases [10, 102]. The ocean, however, exhibits a vast potential for phytate hydrolysis, as evidenced by the abundance and widespread geographic distribution of phytases genes (Fig. 3). Although BPP was historically considered as the primary phytase group in aquatic environments [41], recent findings reveal the widespread occurrence and enzymatic activities of acidic and atypical phytases under alkaline conditions. These expands our understanding of phytate hydrolysis in marine environments (Fig. 7). Moreover, the convergent evolution of these phytases, including functional specialization in atypical phytases, strongly suggests that phytate hydrolysis may have substantial evolutionary importance. Phosphorus is an essential macronutrient for microorganisms [6, 103]. DIP derived from the degradation of phytate by phytases possibly serves as a valuable nutritional resource for microbial growth, potentially promoting the prevalence of Proteobacteria, which are likely the primary sources of phytase in marine environments (Fig. 4). Moreover, the presence of gene sequences of phytases in anaerobic bacteria, such as Thermoanaerobaculia [104] and certain Deltaproteobacteria [104, 105], also demonstrates a wide range of biological and environmental adaptability of this phytase-mediated phosphorus-producing process.

Apart from its role in phosphorus nutrition, phytate and the other InsPs, such as the second messenger Ins(1,4,5)P_3_, are also involved in various physiological processes in plants and animals, including signal transduction [106, 107] and energy metabolism [108]. Similarly, the ultimate catabolite of BPP phytases, Ins(2,4,6)P_3_, may also function as a signaling molecule influencing diverse aspects of cell biology. In marine ecosystems, the DIP released through extracellular InsPs hydrolysis serves as “public goods,” benefiting the entire microbial community. This shared resource could influence microbial community composition, ecosystem structure, and functional traits, especially under conditions of phosphorus limitation [42, 77]. These findings underscore the need for further comprehensive investigations into the physiological, biochemical, and ecological roles of InsPs and their degradation products. Such research will help unravel their significance in marine environments, providing insights into their impact on nutrient cycling, microbial interactions, and ecosystem resilience under environmental stress.

## Supplementary Material

supplementary_figures_wraf161

Table_S1_wraf161

Supplementary_Table_S2_wraf161

Table_S3_wraf161

Supplementary_Table_S4_wraf161

Supplementary_Table_S5_wraf161

Supplementary_Table_S6_wraf161

## Data Availability

All the polar metagenomic datasets have been deposited in the NCBI database (BioProject accession no. PRJNA588686). All information is included in the manuscript or supplementary files.

## References

[1] Duhamel S, Diaz JM, Adams JC. et al. Phosphorus as an integral component of global marine biogeochemistry. *Nat Geosci* 2021;14:359–68. 10.1038/s41561-021-00755-8

[2] Liang Z, Letscher RT, Knapp AN. Dissolved organic phosphorus concentrations in the surface ocean controlled by both phosphate and iron stress. *Nat Geosci* 2022;15:651–7. 10.1038/s41561-022-00988-1

[3] Jin H, Zhang C, Meng S. et al. Atmospheric deposition and river runoff stimulate the utilization of dissolved organic phosphorus in coastal seas. *Nat Commun* 2024;15:658. 38291022 10.1038/s41467-024-44838-7 PMC10828365

[4] Reynolds S, Mahaffey C, Roussenov V. et al. Evidence for production and lateral transport of dissolved organic phosphorus in the eastern subtropical North Atlantic. *Global Biogeochem Cy* 2014;28:805–24. 10.1002/2013GB004801

[5] Kolowith LC, Ingall ED, Benner R. Composition and cycling of marine organic phosphorus. *Limnol Oceanogr* 2001;46:309–20. 10.4319/lo.2001.46.2.0309

[6] Tyrrell T . The relative influences of nitrogen and phosphorus on oceanic primary production. *Nature.* 1999;400:525–31.

[7] Liu X, Han R, Cao Y. et al. Enhancing phytate availability in soils and phytate-P acquisition by plants: a review. *Environ Sci Technol* 2022;56:9196–219. 10.1021/acs.est.2c00099 35675210 PMC9261192

[8] Lei XG, Porres JM, Mullaney EJ., et al. Phytase: Source, structure and application. In: Polaina J, MacCabe AP (eds.). Industrial Enzymes: Structure, Function and Applications, Dordrecht: Springer Netherlands, Berlin, Germany. 17:505–29. 10.4240/wjgs.v17.i7.100766

[9] Scott BM, Koh K, Rix GD. Structural and functional profile of phytases across the domains of life. *Curr Res Struct Biol* 2024;7:100139. 10.1016/j.crstbi.2024.100139 38562944 PMC10982552

[10] Turner BL, Papházy MJ, Haygarth PM. et al. Inositol phosphates in the environment. *Philos Trans R Soc Lond Ser B Biol Sci* 2002;357:449–69. 10.1098/rstb.2001.0837 12028785 PMC1692967

[11] Yao MZ, Zhang YH, Lu WL. et al. Phytases: crystal structures, protein engineering and potential biotechnological applications. *J Appl Microbiol* 2012;112:1–14. 10.1111/j.1365-2672.2011.05181.x 22017627

[12] Kumar V, Yadav AN, Verma P. et al. Β-propeller phytases: diversity, catalytic attributes, current developments and potential biotechnological applications. *Int J Biol Macromol* 2017;98:595–609. 10.1016/j.ijbiomac.2017.01.134 28174082

[13] Puhl AA, Gruninger RJ, Greiner R. et al. Kinetic and structural analysis of a bacterial protein tyrosine phosphatase-like *myo*-inositol polyphosphatase. *Protein Sci* 2007;16:1368–78. 10.1110/ps.062738307 17567745 PMC2206706

[14] Mullaney EJ, Daly CB, Ullah AH. Advances in phytase research. Adv Appl Microbiol 2000;47:157–99, San Diego, Calif., Elsevier, 10.1016/S0065-2164(00)47004-8 12876797

[15] Schenk G, Mitić N, Hanson GR. et al. Purple acid phosphatase: a journey into the function and mechanism of a colorful enzyme. *Coordin Chem Rev* 2013;257:473–82. 10.1016/j.ccr.2012.03.020

[16] Tan H, Mooij MJ, Barret M. et al. Identification of novel phytase genes from an agricultural soil-derived metagenome. *J Microbiol Biotechnol* 2014;24:113–8. 10.4014/jmb.1307.07007 24150499

[17] Castillo Villamizar GA, Funkner K, Nacke H. et al. Functional metagenomics reveals a new catalytic domain, the metallo-β-lactamase superfamily domain, associated with phytase activity. *mSphere.* 2019;4:e00167–19. 10.1128/mSphere.00167-19 31217298 PMC6584368

[18] Castillo Villamizar GA, Nacke H, Boehning M. et al. Functional metagenomics reveals an overlooked diversity and novel features of soil-derived bacterial phosphatases and phytases. *mBio.* 2019;10:e01966–18. 10.1128/mBio.01966-18 30696742 PMC6355987

[19] Greiner R, Carlsson N, Alminger ML. Stereospecificity of myo-inositol hexakisphosphate dephosphorylation by a phytate-degrading enzyme of *Escherichia coli*. *J Biotechnol* 2001;84:53–62. 10.1016/s0168-1656(00)00331-x 11035187

[20] Ragon M, Aumelas A, Chemardin P. et al. Complete hydrolysis of *myo*-inositol hexakisphosphate by a novel phytase from *Debaryomyces castellii* CBS 2923. *Appl Microbiol Biotechnol* 2008;78:47–53. 10.1007/s00253-007-1275-3 18046551

[21] Greiner R, Larsson Alminger M, Carlsson NG. et al. Pathway of dephosphorylation of *myo*-inositol hexakisphosphate by phytases of legume seeds. *J Agric Food Chem* 2002;50:6865–70. 10.1021/jf025620t 12405789

[22] Puhl AA, Greiner R, Selinger LB. A protein tyrosine phosphatase-like inositol polyphosphatase from *Selenomonas ruminantium* subsp. lactilytica has specificity for the 5-phosphate of *myo*-inositol hexakisphosphate. *Int J Biochem Cell Biol* 2008;40:2053–64. 10.1016/j.biocel.2008.02.003 18358762

[23] Puhl AA, Greiner R, Selinger LB. Stereospecificity of *myo*-inositol hexakisphosphate hydrolysis by a protein tyrosine phosphatase-like inositol polyphosphatase from *Megasphaera elsdenii*. *Appl Microbiol Biotechnol* 2009;82:95–103. 10.1007/s00253-008-1734-5 18853154

[24] Greiner R, Lim BL, Cheng C. et al. Pathway of phytate dephosphorylation by beta-propeller phytases of different origins. *Can J Microbiol* 2007;53:488–95. 10.1139/W07-015 17612603

[25] Suzumura M, Kamatani A. Origin and distribution of inositol hexaphosphate in estuarine and coastal sediments. *Limnol Oceanogr* 1995;40:1254–61. 10.4319/lo.1995.40.7.1254

[26] Suzumura M, Kamatani A. Mineralization of inositol hexaphosphate in aerobic and anaerobic marine sediments: implications for the phosphorus cycle. *Geochim Cosmochim Ac* 1995;59:1021–6. 10.1016/0016-7037(95)00006-2

[27] Li J, Zhang K, Lin X. et al. Phytate as a phosphorus nutrient with impacts on iron stress-related gene expression for phytoplankton: insights from the diatom *Phaeodactylum tricornutum*. *Appl Environ Microbiol* 2022;88:e0209721. 10.1128/AEM.02097-21 34757820 PMC8788711

[28] Wilson MS, Bulley SJ, Pisani F. et al. A novel method for the purification of inositol phosphates from biological samples reveals that no phytate is present in human plasma or urine. *Open Biol* 2015;5:150014. 10.1098/rsob.150014 25808508 PMC4389793

[29] Feng W, Wu F, He Z. et al. Simulated bioavailability of phosphorus from aquatic macrophytes and phytoplankton by aqueous suspension and incubation with alkaline phosphatase. *Sci Total Environ* 2018;616-617:1431–9. 10.1016/j.scitotenv.2017.10.172 29074246

[30] Lin L, Ling J, Peng Q. et al. The distribution characteristics of β-propeller phytase genes in rhizosphere sediment provide insight into species specialty from phytic mineralization in subtropical and tropical seagrass ecosystems. *Ecotoxicology.* 2021;30:1781–8. 10.1007/s10646-021-02425-2 34115256

[31] Kumar A, Singh B, Raigond P. et al. Phytic acid: blessing in disguise, a prime compound required for both plant and human nutrition. *Food Res Int* 2021;142:110193. 10.1016/j.foodres.2021.110193 33773669

[32] Kumar V, Sinha AK, Makkar HPS. et al. Dietary roles of phytate and phytase in human nutrition: a review. *Food Chem* 2010;120:945–59. 10.1016/j.foodchem.2009.11.052

[33] Balwani I, Chakravarty K, Gaur S. Role of phytase producing microorganisms towards agricultural sustainability. *Biocatal Agric Biotechnol* 2017;12:23–9. 10.1016/j.bcab.2017.08.010

[34] Madsen CK, Brinch-Pedersen H. Molecular advances on phytases in barley and wheat. *Int J Mol Sci* 2019;20:2459. 10.3390/ijms20102459 31109025 PMC6566229

[35] Dersjant-Li Y, Awati A, Schulze H. et al. Phytase in non-ruminant animal nutrition: a critical review on phytase activities in the gastrointestinal tract and influencing factors. *J Sci Food Agric* 2015;95:878–96. 10.1002/jsfa.6998 25382707 PMC4368368

[36] Joudaki H, Aria N, Moravej R. et al. Microbial phytases: properties and applications in the food industry. *Curr Microbiol* 2023;80:374. 10.1007/s00284-023-03471-1 37847302 PMC10581959

[37] Herrmann KR, Ruff AJ, Infanzón B. et al. Engineered phytases for emerging biotechnological applications beyond animal feeding. *Appl Microbiol Biotechnol* 2019;103:6435–48. 10.1007/s00253-019-09962-1 31254000

[38] Li J, Zhang K, Li L. et al. Two-sided effects of the organic phosphorus phytate on a globally important marine coccolithophorid phytoplankton. *Microbiol Spectr* 2023;11:e0125523. 10.1128/spectrum.02806-23 37702480 PMC10655706

[39] Zheng S, Wang B, Xu G. et al. Effects of organic phosphorus on methylotrophic methanogenesis in coastal lagoon sediments with seagrass (*Zostera marina*) colonization. *Front Microbiol* 2020;11:1770. 10.3389/fmicb.2020.01770 32849394 PMC7411354

[40] Suliasih S, Widawati S, Nadhirah A. et al. Phytase activity of phytase-producing bacteria isolated from mangrove sediment. *IOP Conf Ser: Earth Environ Sci* 2022;976:012041. 10.1088/1755-1315/976/1/012041

[41] Lim BL, Yeung P, Cheng C. et al. Distribution and diversity of phytate-mineralizing bacteria. *ISME J* 2007;1:321–30. 10.1038/ismej.2007.40 18043643

[42] Moore CM, Mills MM, Arrigo KR. et al. Processes and patterns of oceanic nutrient limitation. *Nat Geosci* 2013;6:701–10. 10.1038/ngeo1765

[43] Burbano C, Muzquiz M, Osagie A. et al. Determination of phytate and lower inositol phosphates in Spanish legumes by HPLC methodology. *Food Chem* 1995;52:321–5. 10.1016/0308-8146(95)92831-4

[44] Costa-Bauza A, Grases F, Gomila I. et al. A simple and rapid colorimetric method for determination of phytate in urine. *Urol Res* 2012;40:663–9. 10.1007/s00240-012-0473-3 22476541

[45] Thompson JD, Higgins DG, Gibson TJ. CLUSTAL W: improving the sensitivity of progressive multiple sequence alignment through sequence weighting, position-specific gap penalties and weight matrix choice. *Nucleic Acids Res* 1994;22:4673–80. 10.1093/nar/22.22.4673 7984417 PMC308517

[46] Hall TA . BioEdit: a user-friendly biological sequence alignment editor and analysis program for windows 95/98/NT. *Nucl Acids Symp Ser* 1999;41:95–8.

[47] Lidbury IDEA, Scanlan DJ, Murphy ARJ. et al. A widely distributed phosphate-insensitive phosphatase presents a route for rapid organophosphorus remineralization in the biosphere. *Proc Natl Acad Sci USA* 2022;119:e2118122119. 10.1073/pnas.2118122119 35082153 PMC8812569

[48] Torcello-Requena A, Murphy ARJ, Lidbury IDEA. et al. A distinct, high-affinity, alkaline phosphatase facilitates occupation of P-depleted environments by marine picocyanobacteria. *Proc Natl Acad Sci USA* 2024;121:e2312892121. 10.1073/pnas.2312892121 38713622 PMC11098088

[49] O'Brie PJ, Herschlag D. Functional interrelationships in the alkaline phosphatase superfamily: phosphodiesterase activity of *Escherichia coli* alkaline phosphatase. *Biochemistry.* 2001;40:5691–9. 11341834 10.1021/bi0028892

[50] Rodriguez F, Lillington J, Johnson S. et al. Crystal structure of the *Bacillus subtilis* phosphodiesterase PhoD reveals an iron and calcium-containing active site. *J Biol Chem* 2014;289:30889–99. 10.1074/jbc.M114.604892 25217636 PMC4223295

[51] Bihani SC, Das A, Nilgiriwala KS. et al. X-ray structure reveals a new class and provides insight into evolution of alkaline phosphatases. *PLoS One* 2011;6:e22767. 10.1371/journal.pone.0022767 21829507 PMC3145672

[52] Yong SC, Roversi P, Lillington J. et al. A complex iron-calcium cofactor catalyzing phosphotransfer chemistry. *Science.* 2014;345:1170–3. 10.1126/science.1254237 25190793 PMC4175392

[53] Teng Z-J, Qin Q-L, Zhang W. et al. Biogeographic traits of dimethyl sulfide and dimethylsulfoniopropionate cycling in polar oceans. *Microbiome.* 2021;9:207. 10.1186/s40168-021-01153-3 34654476 PMC8520302

[54] Sunagawa S, Acinas SG, Bork P. et al. *Tara oceans*: towards global ocean ecosystems biology. *Nat Rev Microbiol* 2020;18:428–45. 10.1038/s41579-020-0364-5 32398798

[55] Chen IMA, Chu K, Palaniappan K. et al. The IMG/M data management and analysis system v.7: content updates and new features. *Nucleic Acids Res* 2023;51:D723–32. 10.1093/nar/gkac976 36382399 PMC9825475

[56] Johnson LS, Eddy SR, Portugaly E. Hidden Markov model speed heuristic and iterative HMM search procedure. *BMC Bioinformatics* 2010;11:431. 10.1186/1471-2105-11-431 20718988 PMC2931519

[57] Edgar RC . MUSCLE: multiple sequence alignment with high accuracy and high throughput. *Nucleic Acids Res* 2004;32:1792–7. 10.1093/nar/gkh340 15034147 PMC390337

[58] Price MN, Dehal PS, Arkin AP. FastTree: computing large minimum evolution trees with profiles instead of a distance matrix. *Mol Biol Evol* 2009;26:1641–50. 10.1093/molbev/msp077 19377059 PMC2693737

[59] He Z, Zhang H, Gao S. et al. Evolview v2: an online visualization and management tool for customized and annotated phylogenetic trees. *Nucleic Acids Res* 2016;44:W236–41. 10.1093/nar/gkw370 27131786 PMC4987921

[60] Sunagawa S, Mende DR, Zeller G. et al. Metagenomic species profiling using universal phylogenetic marker genes. *Nat Methods* 2013;10:1196–9. 10.1038/nmeth.2693 24141494

[61] Dumon-Seignovert L, Cariot G, Vuillard L. The toxicity of recombinant proteins in *Escherichia coli*: a comparison of overexpression in BL21(DE3), C41(DE3), and C43(DE3). *Protein Expr Purif* 2004;37:203–6. 10.1016/j.pep.2004.04.025 15294299

[62] Nielsen H, Tsirigos KD, Brunak S. et al. A brief history of protein sorting prediction. *Protein J* 2019;38:200–16. 10.1007/s10930-019-09838-3 31119599 PMC6589146

[63] Walker JM . The bicinchoninic acid (BCA) assay for protein quantitation. *Methods Mol Biol* 1994;32:5–8. 10.1385/0-89603-268-X:5 7951748

[64] Borgi MA, Boudebbouze S, Aghajari N. et al. The attractive recombinant phytase from *bacillus licheniformis*: biochemical and molecular characterization. *Appl Microbiol Biotechnol* 2014;98:5937–47. 10.1007/s00253-013-5421-9 24337251

[65] Sambo F, Finotello F, Lavezzo E. et al. Optimizing PCR primers targeting the bacterial 16S ribosomal RNA gene. *BMC Bioinformatics* 2018;19:343. 30268091 10.1186/s12859-018-2360-6 PMC6162885

[66] Altschul SF, Gish W, Miller W. et al. Basic local alignment search tool. *J Mol Biol* 1990;215:403–10. 10.1016/S0022-2836(05)80360-2 2231712

[67] Ihaka R, Gentleman R. R: a language for data analysis and graphics. *J Comput Graph Stat* 1996;5:299–314. 10.1080/10618600.1996.10474713

[68] Villanueva RAM, Chen ZJ. ggplot2: elegant graphics for data analysis (2nd ed.). Meas-Interdiscip Res 2019;17:160–7.

[69] Gao C-H, Chen C, Akyol T. et al. ggVennDiagram: intuitive Venn diagram software extended. *iMeta.* 2024;3:e177. 10.1002/imt2.177 38868514 PMC10989133

[70] Dixon P . VEGAN, a package of R functions for community ecology. *J Veg Sci* 2003;14:927–30. 10.1111/j.1654-1103.2003.tb02228.x

[71] Brunson JC . Ggalluvial: layered grammar for alluvial plots. *J Open Source Softw* 2020;5:2017. 10.21105/joss.02017 36919162 PMC10010671

[72] Gu Z, Gu L, Eils R. et al. Circlize implements and enhances circular visualization in R. *Bioinformatics.* 2014;30:2811–2. 10.1093/bioinformatics/btu393 24930139

[73] Ning D, Deng Y, Tiedje JM. et al. A general framework for quantitatively assessing ecological stochasticity. *Proc Natl Acad Sci USA* 2019;116:16892–8. 10.1073/pnas.1904623116 31391302 PMC6708315

[74] Schlitzer R . Interactive analysis and visualization of geoscience data with ocean data view. *Comput Geosci-UK* 2002;28:1211–8. 10.1016/S0098-3004(02)00040-7

[75] Kumar S, Nei M, Dudley J. et al. MEGA: a biologist-centric software for evolutionary analysis of DNA and protein sequences. *Brief Bioinform* 2008;9:299–306. 10.1093/bib/bbn017 18417537 PMC2562624

[76] McIntyre CA, Arthur CJ, Evershed RP. High-resolution mass spectrometric analysis of *myo*-inositol hexakisphosphate using electrospray ionisation orbitrap. *Rapid Commun Mass Spectrom* 2017;31:1681–9. 10.1002/rcm.7935 28696018 PMC5639359

[77] Ustick LJ, Larkin AA, Garcia CA. et al. Metagenomic analysis reveals global-scale patterns of ocean nutrient limitation. *Science.* 2021;372:287–91. 33859034 10.1126/science.abe6301

[78] Shin S, Ha NC, Oh BC. et al. Enzyme mechanism and catalytic property of beta propeller phytase. *Structure.* 2001;9:851–8. 10.1016/S0969-2126(01)00637-2 11566134

[79] Lim D, Golovan S, Forsberg CW. et al. Crystal structures of *Escherichia coli* phytase and its complex with phytate. *Nat Struct Biol* 2000;7:108–13. 10.1038/72371 10655611

[80] Gruninger RJ, Thibault J, Capeness MJ. et al. Structural and biochemical analysis of a unique phosphatase from *Bdellovibrio bacteriovorus* reveals its structural and functional relationship with the protein tyrosine phosphatase class of phytase. *PLoS One* 2014;9:e94403. 10.1371/journal.pone.0094403 24718691 PMC3981807

[81] Sunagawa S, Mende DR, Zeller G. et al. Metagenomic species profiling using universal phylogenetic marker genes. *Nat Methods* 2013;10:1196–9. 10.1038/nmeth.2693 24141494

[82] Mather RL, Reynolds SE, Wolff GA. et al. Phosphorus cycling in the north and South Atlantic Ocean subtropical gyres. *Nat Geosci* 2008;1:439–43. 10.1038/ngeo232

[83] Matthews TJ, Whittaker RJ. Neutral theory and the species abundance distribution: recent developments and prospects for unifying niche and neutral perspectives. *Ecol Evol* 2014;4:2263–77. 10.1002/ece3.1092 25360266 PMC4201439

[84] Zhou J, Ning D. Stochastic community assembly: does it matter in microbial ecology? *Microbiol Mol Biol R* 2017;81:e00002–17. 10.1128/MMBR.00002-17 PMC5706748 29021219

[85] Gravel D, Canham CD, Beaudet M. et al. Reconciling niche and neutrality: the continuum hypothesis. *Ecol Lett* 2006;9:399–409. 10.1111/j.1461-0248.2006.00884.x 16623725

[86] Zhou J, Deng Y, Zhang P. et al. Stochasticity, succession, and environmental perturbations in a fluidic ecosystem. *Proc Natl Acad Sci USA* 2014;111:E836–45. 10.1073/pnas.1324044111 24550501 PMC3948316

[87] Zhou J, Qin W, Lu X. et al. The diversity and ecological significance of microbial traits potentially involved in B12 biosynthesis in the global ocean. *mlife.* 2023;2:416–27. 10.1002/mlf2.12095 38818271 PMC10989127

[88] Fang W, Fan T, Wang S. et al. Seasonal changes driving shifts in microbial community assembly and species coexistence in an urban river. *Sci Total Environ* 2023;905:167027. 10.1016/j.scitotenv.2023.167027 37717779

[89] Chave J . Neutral theory and community ecology. *Ecol Lett* 2004;7:241–53. 10.1111/j.1461-0248.2003.00566.x

[90] Tian J, Liao H. The role of intracellular and secreted purple acid phosphatases in plant phosphorus scavenging and recycling. In: Plaxton W.C., Lambers H. (eds.), Annual Plant Reviews. Vol. 48. Phosphorus Metabolism in Plants. 265–287. Hoboken: John Wiley & Sons, 2015, 10.1002/9781118958841.ch10.

[91] Lung S-C, Leung A, Kuang R. et al. Phytase activity in tobacco (*Nicotiana tabacum*) root exudates is exhibited by a purple acid phosphatase. *Phytochemistry.* 2008;69:365–73. 10.1016/j.phytochem.2007.06.036 17897689

[92] White PJ, Veneklaas EJ. Nature and nurture: the importance of seed phosphorus content. *Plant Soil* 2012;357:1–8. 10.1007/s11104-012-1128-4

[93] Lazali M, Louadj L, Ounane G. et al. Localization of phytase transcripts in germinating seeds of the common bean (*Phaseolus vulgaris* L.). *Planta.* 2014;240:471–8. 10.1007/s00425-014-2101-7 24912928

[94] Short F, Carruthers T, Dennison W. et al. Global seagrass distribution and diversity: a bioregional model. *J Exp Mar Biol Ecol* 2007;350:3–20. 10.1016/j.jembe.2007.06.012

[95] UNEP-WCMC, Short F. Global Distribution of Seagrasses (Version 7.1). Seventh Update to the Data Layer Used in Green and Short (2003). Cambridge (UK): UN Environment World Conservation Monitoring Centre, 2021.

[96] Fourqurean JW, Duarte CM, Kennedy H. et al. Seagrass ecosystems as a globally significant carbon stock. *Nat Geosci* 2012;5:505–9. 10.1038/ngeo1477

[97] Duarte CM, Middelburg JJ, Caraco N. Major role of marine vegetation on the oceanic carbon cycle. *Biogeosciences.* 2005;2:1–8. 10.5194/bg-2-1-2005

[98] Krause-Jensen D, Duarte CM. Substantial role of macroalgae in marine carbon sequestration. *Nat Geosci* 2016;9:737–42. 10.1038/ngeo2790

[99] Ortega A, Geraldi NR, Alam I. et al. Important contribution of macroalgae to oceanic carbon sequestration. *Nat Geosci* 2019;12:748–54. 10.1038/s41561-019-0421-8

[100] Filbee-Dexter K, Pessarrodona A, Pedersen MF. et al. Carbon export from seaweed forests to deep ocean sinks. *Nat Geosci* 2024;17:552–9. 10.1038/s41561-024-01449-7

[101] Letscher RT, Primeau F, Moore JK. Nutrient budgets in the subtropical ocean gyres dominated by lateral transport. *Nat Geosci* 2016;9:815–9. 10.1038/ngeo2812

[102] Karl DM . Microbially mediated transformations of phosphorus in the sea: new views of an old cycle. *Annu Rev Mar Sci* 2014;6:279–337. 10.1146/annurev-marine-010213-135046 24405427

[103] Van Mooy BA, Krupke A, Dyhrman ST. et al. Major role of planktonic phosphate reduction in the marine phosphorus redox cycle. *Science.* 2015;348:783–5. 25977548 10.1126/science.aaa8181

[104] Flieder M, Buongiorno J, Herbold CW. et al. Novel taxa of Acidobacteriota implicated in seafloor sulfur cycling. *ISME J* 2021;15:3159–80. 10.1038/s41396-021-00992-0 33981000 PMC8528874

[105] Langwig MV, De Anda V, Dombrowski N. et al. Large-scale protein level comparison of deltaproteobacteria reveals cohesive metabolic groups. *ISME J* 2022;16:307–20. 10.1038/s41396-021-01057-y 34331018 PMC8692467

[106] Prasad A, Jia Y, Chakraborty A. et al. Inositol hexakisphosphate kinase 1 regulates neutrophil function in innate immunity by inhibiting phosphatidylinositol-(3,4,5)-trisphosphate signaling. *Nat Immunol* 2011;12:752–60. 10.1038/ni.2052 21685907 PMC3140608

[107] Irvine RF, Schell MJ. Back in the water: the return of the inositol phosphates. *Nat Rev Mol Cell Bio* 2001;2:327–38. 10.1038/35073015 11331907

[108] Chatree S, Thongmaen N, Tantivejkul K. et al. Role of inositols and inositol phosphates in energy metabolism. *Molecules.* 2020;25:5079. 10.3390/molecules25215079 33139672 PMC7663797

