# Supplementary material for: Inositol phosphates as an overlooked phosphorous source in marine ecosystems
Source: ISME J. 2025 Jul 11;19(1):wraf161. doi: 10.1093/ismejo/wraf161 (PMC12406696; doi:10.1093/ismejo/wraf161)
Supplement: supplementary_figures_wraf161 [file supplementary_figures_wraf161.docx]

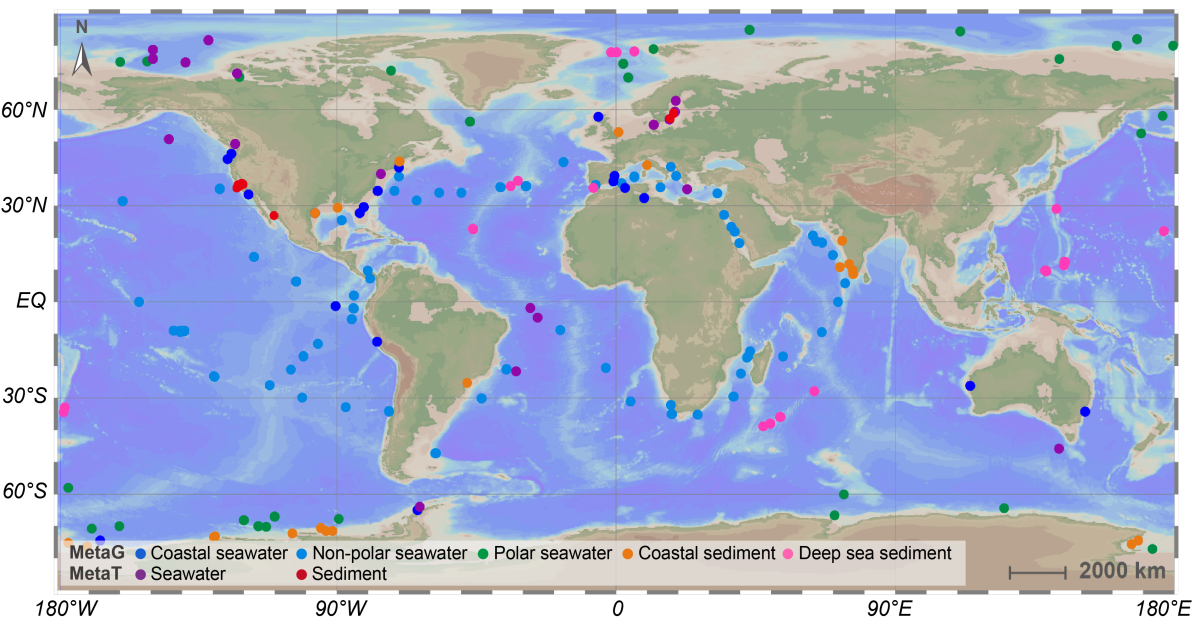


**Figure S1.** Geographic distribution of the metagenomic and metatranscriptomic samples analyzed in this study. MetaG, metagenomes; MetaT, metatranscriptomes.

**
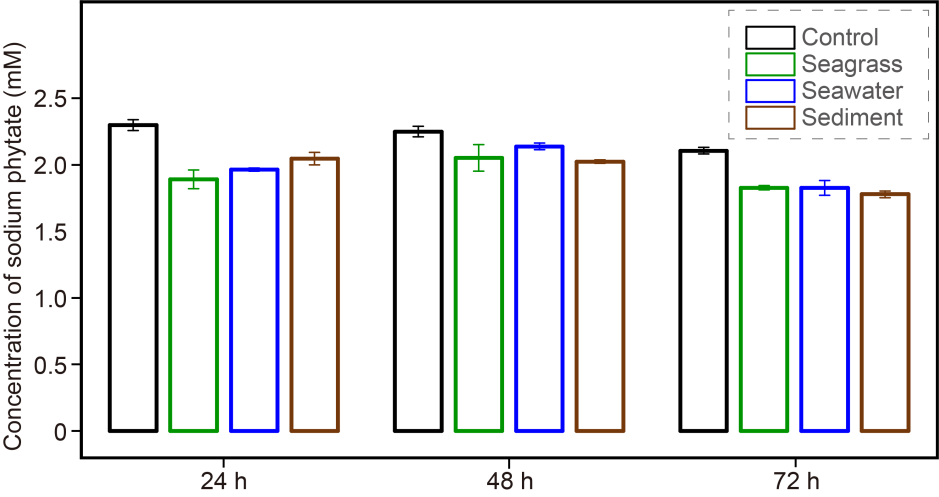
**

**Figure S2.** Phytate degradation capabilities of coastal microbiota from the seagrass phyllosphere, seawater, and sediment off the coast of Qingdao, China. A control group was included without adding biological samples.


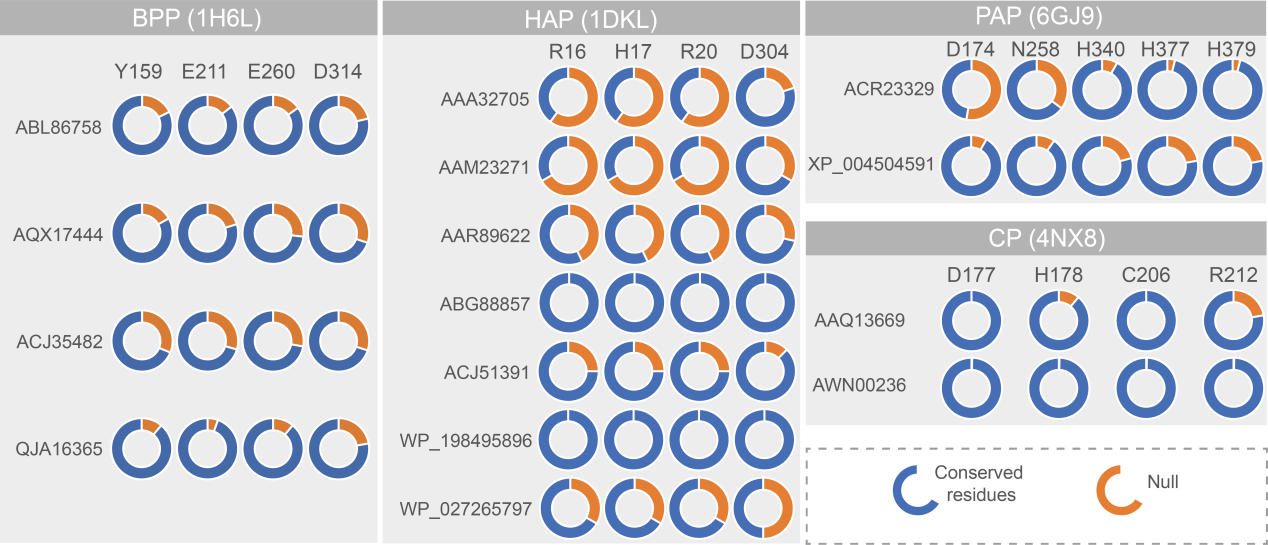


**Figure S3.** Analysis of conserved amino acid residues involved in substrate binding and catalysis of phytases to demonstrate the conservation of retrieved environmental sequences. The amino acid residues suffixed with numbers represent the conserved sites of each protein. The pie charts in each panel show the amino acid compositions of corresponding conserved sites in predicted hits. The key conserved residues of each protein are shown in blue. “Null” indicates the absence of corresponding amino acid residue from environmental hits owing to their short sequence length shown in orange. The structure accession numbers of corresponding proteins in the Protein Data Bank (PDB) are shown in brackets.


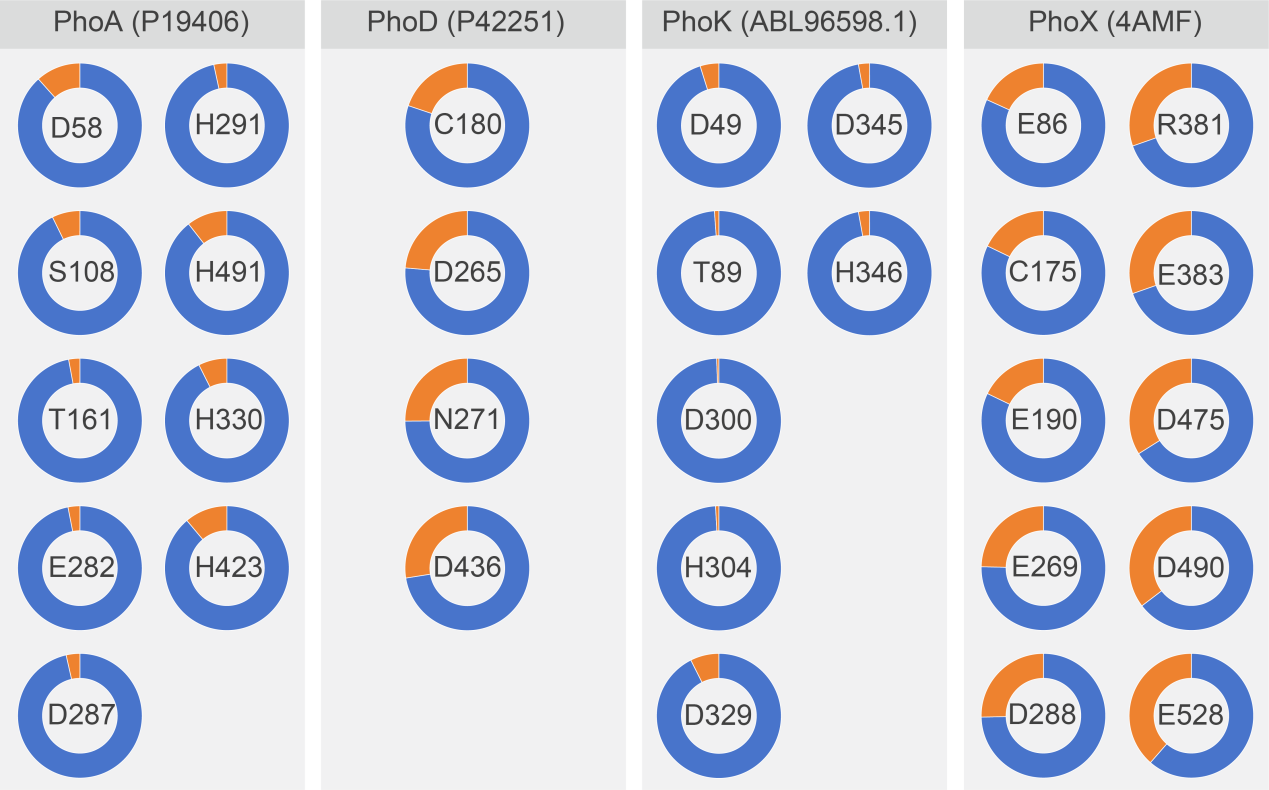


**Figure S4.** Analysis of conserved amino acid residues of alkaline phosphatases involved in metal ion binding to demonstrate the conservation of retrieved environmental sequences. The key conserved residues of each protein are shown in blue. The absence of corresponding amino acid residues from environmental hits owing to their short sequence length is shown in orange. The accession numbers of corresponding proteins in NCBI are shown in brackets.


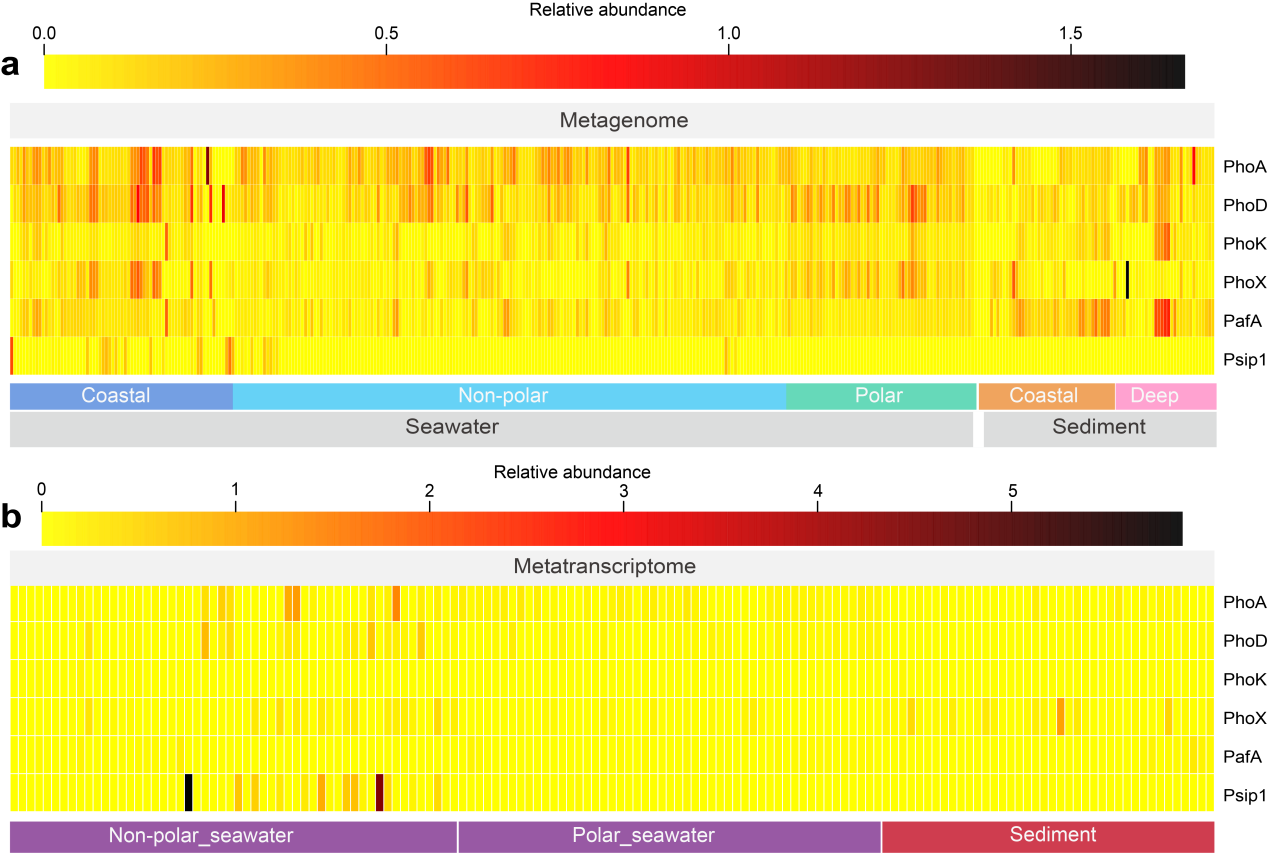


**Figure S5.** The relative abundances of the genetic potential of alkaline phosphatases from (**a**) metagenomic samples and (**b**) metatranscriptomic samples.

**
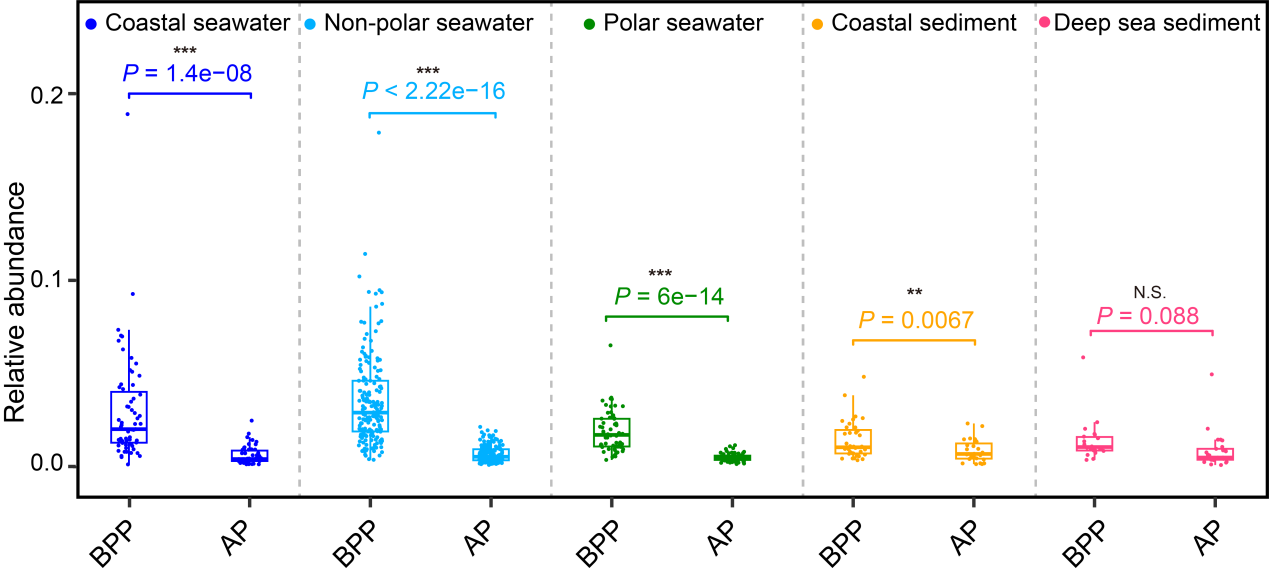
**

**Figure S6.** The comparisons of relative abundances of alkaline phytases (BPP), acid phytases (CP, HAP and PAP). AP, acid phytases. ***, P value < 0.001; **, P value < 0.01; N.S. not significant.


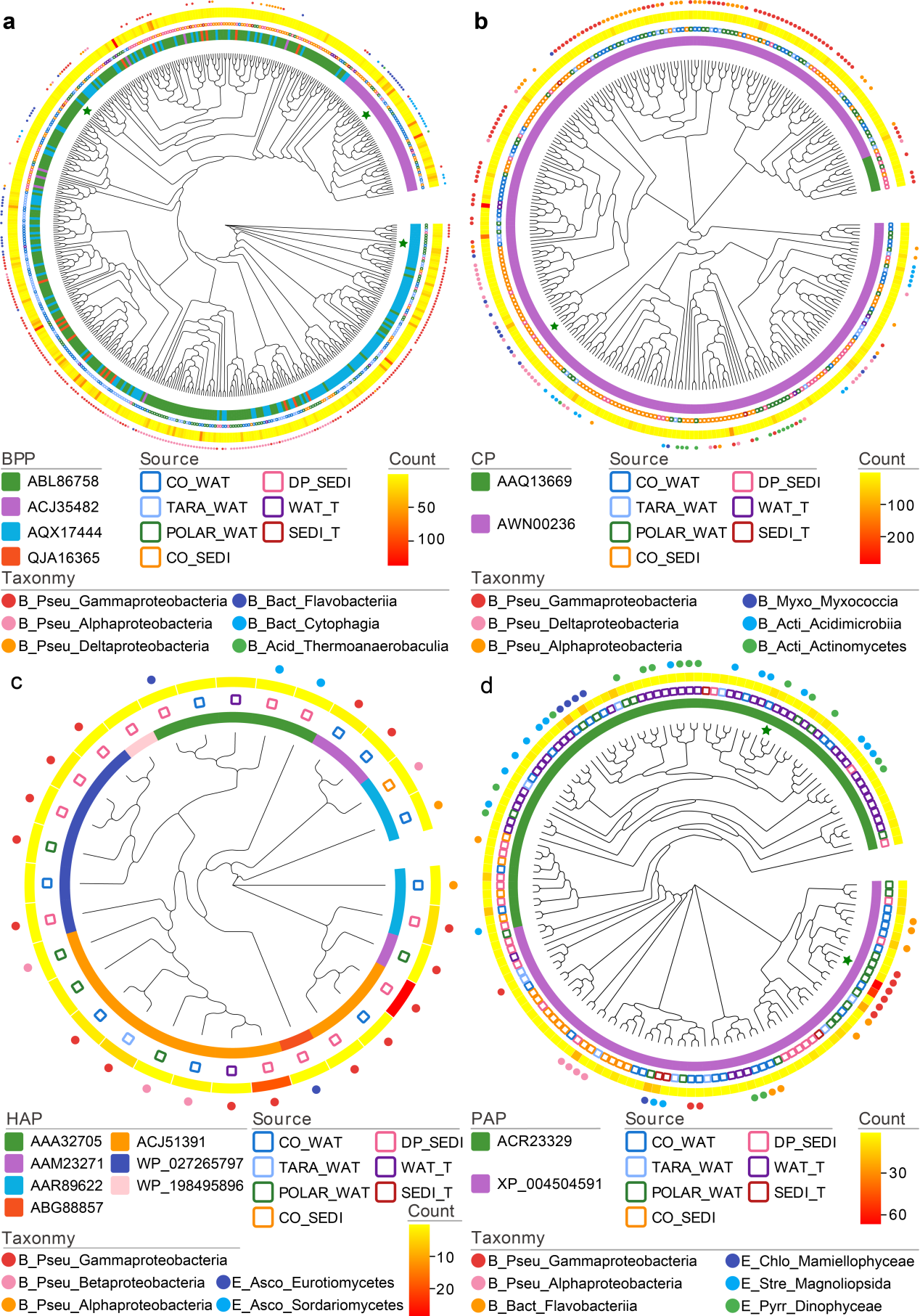


**Figure S7.** The taxonomic composition of typical phytase potentials derived from metagenomic and metatranscriptomic samples. The phylogenetic trees of (**a**) BPP, (**b**) CP, (**c**) HAP and (**d**) PAP were constructed based on their respective genetic potentials. The sequences with similarities exceeding 60% were grouped into one leaf, and the heatmaps indicated the number of sequences in each cluster. B, Bacteria; E, Eukaryota; Pseu, Pseudomonadota; Bact, Bacteroidota; Acti, Actinomycetota; Myxo, Myxococcota; Acid, Acidobacteriota; Asco, Ascomycota; Ther, Thermodesulfobacteriota; Chlo, Chloroflexota; Stre, Streptophyta; Pyrr, Pyrrophyta; Gemm, Gemmatimonadota.


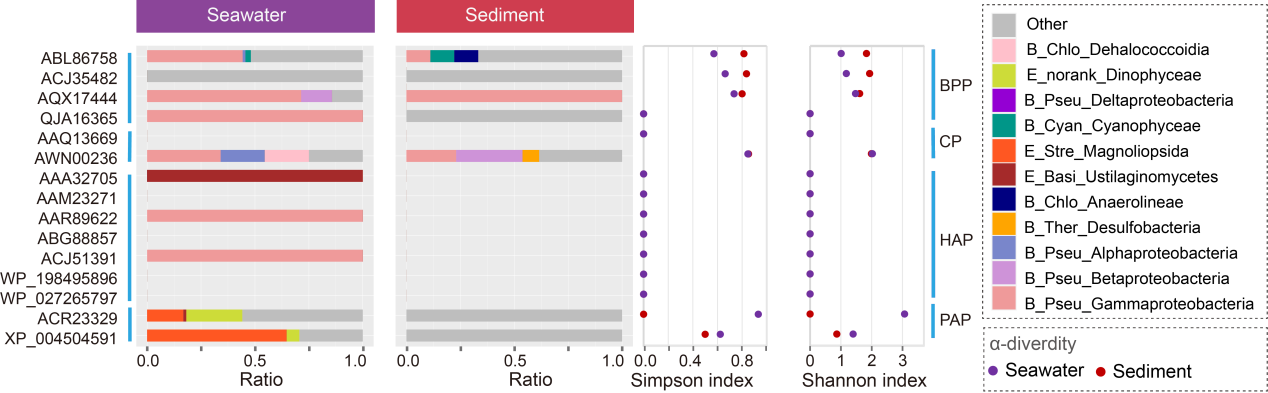


**Figure S8.** The taxonomic composition of genetic potentials from marine metatranscriptomic samples involved in the breakdown of phytate. The relative abundances of each protein in all the metatranscriptomes were summarized using box plots, and the heatmap demonstrating the frequency of samples with detected transcript potentials was shown on the right side. The taxonomic compositions of phytases in metatranscriptomic samples at the class level were visualized by stack maps. B, Bacteria; E, Eukaryota; Pseu, Pseudomonadota; Cyan, Cyanobacteriota; Stre, Streptophyta; Basi, Basidiomycota; Chlo, Chloroflexota; Ther, Thermodesulfobacteriota. The α-diversities of phytases from seawater and sediment samples were indicated by purple and red dots, respectively.


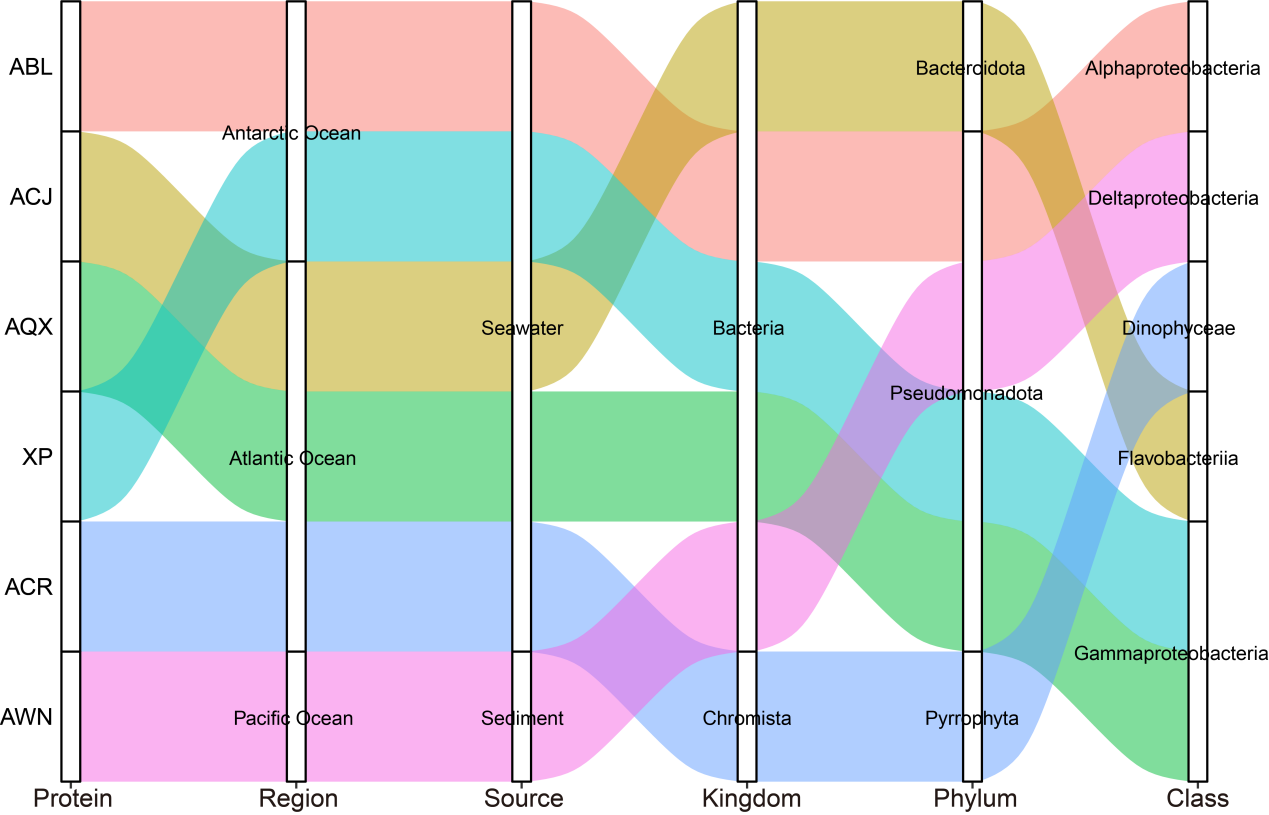


**Figure S9.** Taxonomic profiling of detected phytase potentials derived from marine metagenomic samples. ABL, ABL86758; ACJ, ACJ35482; AQX, AQX17444; XP, XP_004504591; ACR, ACR23329; AWN, AWN00236.

**
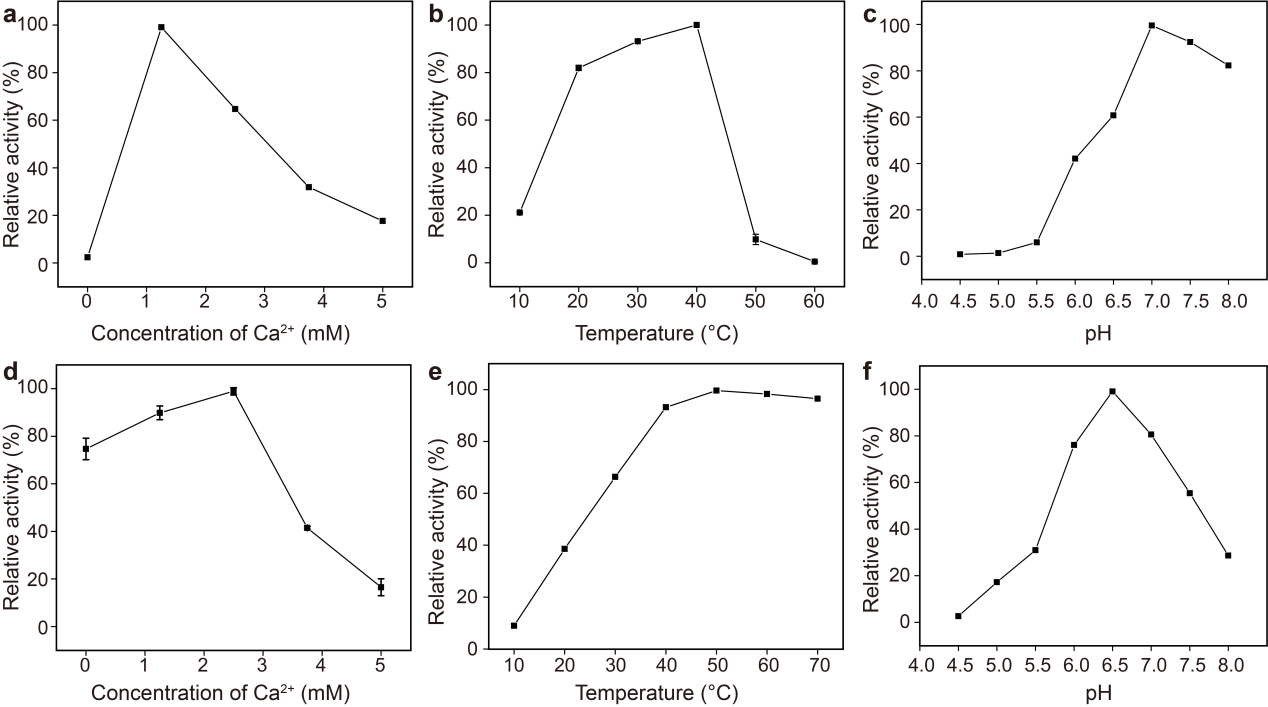
**

**Figure S10.** Biochemical properties of purified phytases from marine environments. **a-c** The effect of (**a**) Ca^2+^ concentration, (**b**) temperature and (**c**) pH on BPP_ABL dephosphorylation activity with InsP_6_ as substrate. The activity of BPP_ABL at 1.25 mM Ca^2+^, 40 °C, pH 7.0 was defined as 100%. **d-f** The effect of (**d**) Ca^2+^ concentration, (**e**) temperature and (**f**) pH on PAP_XP dephosphorylation activity with InsP_6_ as substrate. The activity of PAP_XP at 2.5 mM Ca^2+^, 50 °C, pH 6.5 was defined as 100%. **g-h** Enzymatic kinetic parameters for InsP_6_ dephosphorylation by (**g**) BPP_ABL and (**h**) PAP_XP.

**
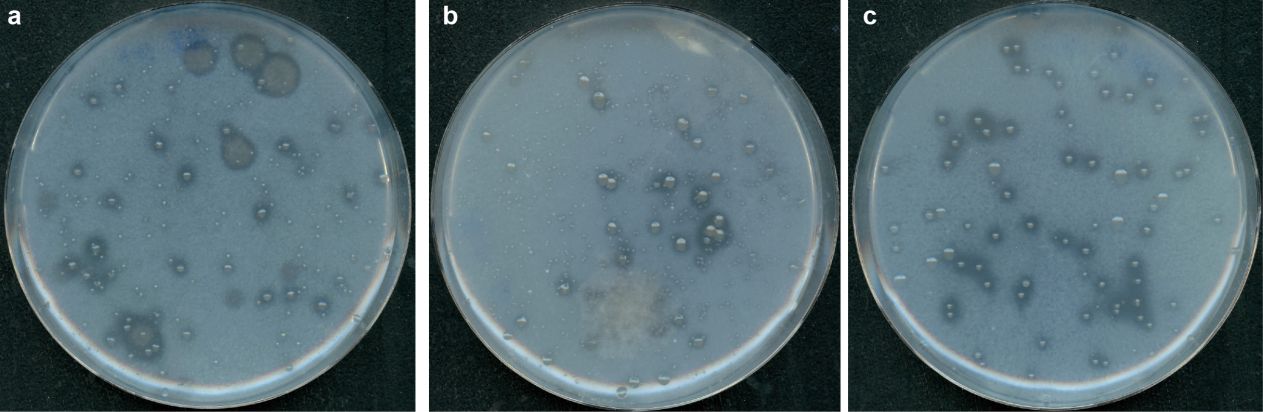
**

**Figure S11.** The phytate degradation capability of microbial consortia from (**a**) seagrass phyllosphere, (**b**) seawater and (**c**) sediment. Microbial strains with phytate hydrolysis abilities were indicated by transparent zones.
