# Supplementary material for: Inositol phosphates as an overlooked phosphorous source in marine ecosystems
Source: ISME J. 2025 Jul 11;19(1):wraf161. doi: 10.1093/ismejo/wraf161 (PMC12406696; doi:10.1093/ismejo/wraf161)
Supplement: Table_S1_wraf161 [file table_s1_wraf161.pdf]

Table S1. The biochemically or structurally information of alkaline phosphates.

Enzyme	Homologs*	Conserved active sites**
PhoA	**P19406**; P00634; P35483	S108; D58; T161; E282; D287; H291; D329; H330; H423
PhoD	**P42251**; BAK26808.1; EFE80684.1	C180; D265; N271; D436
PhoK	**ABL96598.1**; 5XWI; 3Q3Q	D49; T89; D300; H304; D345; H346; H491
PhoX	**4AMF**; XHO88174.1;WP_025956122.1	E86; C175; E190; E269; D288; R381; E383; D475; D490; E528
PafA	WP_407482967.1, WP_012788398.1, WP_148230523.1, WP_289878372.1	-
Psip1	WP_011133045.1, WP_201737941.1, WP_225867717.1	-

* Sequences were retrieved from the National Center for Biotechnology Information (NCBI) database or the Protein Data Bank (PDB) database. The representative sequences are highlighted in bold.

** Conserved active sites are involved in metal ion binding.
