# Supplementary material for: Inositol phosphates as an overlooked phosphorous source in marine ecosystems
Source: ISME J. 2025 Jul 11;19(1):wraf161. doi: 10.1093/ismejo/wraf161 (PMC12406696; doi:10.1093/ismejo/wraf161)
Supplement: Table_S3_wraf161 [file table_s3_wraf161.pdf]

Table S3. The qPCR primers used in this study.

Name*	Forward Primer	Reverse Primer
BPP_ABL	TGCTTCATTCCTATCCTACC	TTCCTTCAGACCGATTGG
BPP_ACJ	CTACCTCCACCAACAATAG	TTATCACTCATCGCAACAA
BPP_AQX	GGAACAACTCAGCGTCAAC	GAGCCTGCCCTGGTAAAG
CP_AWN	AACAGTACATGGAAGAGATGGAAC	GTCAGCAGCAAGGCGAAG
PAP_ACR	ATCTGTATCAAGGTGCTGTC	AAGTATGCCGCTATGCTC
PAP_XP	ATGACTACAGAGGTGATG	TTCTACGAATGGAGTTGT
16S_V6	CAACGCGAAGAACCTTACC	CGACAGCCATGCANCACCT

* BPP, β-propeller phytases, represented by three distinct clades (ABL, ACJ, AQX); PAP, purple acid phytases represented by the XP clade; CP, cysteine phytases represented by the AWN clade.
